# Predicting depressive and anxiety symptoms among Lebanese and Syrian adults in a suburb of Beirut, Lebanon, during concurrent crises: nested cross-sectional study

**DOI:** 10.1136/bmjopen-2025-101258

**Published:** 2026-05-11

**Authors:** Hazar Shamas, Marie-Elizabeth Ragi, Berthe Abi Zeid, Jocelyn DeJong, Stephen J McCall, CAEP Study Group, Aline Germani

**Affiliations:** 1 Center for Research on Population and Health, Faculty of Health Sciences, American University of Beirut, Beirut, Lebanon; 2 Department of Epidemiology and Population Health, Faculty of Health Sciences, American University of Beirut, Beirut, Lebanon; 3 Faculty of Health Sciences, American University of Beirut, Beirut, Lebanon

**Keywords:** Epidemiology, Refugees, Machine Learning, Depression & mood disorders, Anxiety disorders

## Abstract

**Abstract:**

**Objective:**

This study aimed to develop prediction models for symptoms of poor mental health among Lebanese adults and adult Syrian refugees or migrants residing in a suburb of Beirut, Lebanon, separately.

**Design:**

Nested cross-sectional study.

**Setting:**

Sin-El-Fil, a suburb east of Beirut, Lebanon.

**Participants:**

Lebanese and Syrian adults residing in low socio-economic status areas of Sin-El-Fil.

**Primary and secondary outcome measures:**

Primary outcome was having depressive symptoms, ascertained through the Patient Health Questionnaire-9 where a total summative score of 10 or more indicated having depressive symptoms. Secondary outcome was having anxiety symptoms, ascertained through the Generalised Anxiety Disorder-7 where a total summative score of 10 or more indicated having anxiety symptoms.

**Results:**

Of 1986 participants, 1322 (66.5%) were Lebanese adults, 664 (33.5%) were Syrian refugees or migrants. Among Lebanese adults and adult Syrian refugees or migrants, 324 (25.3%) and 289 (43.9%) had depressive symptoms, respectively. Having pain that impacts usual activity, having debt, not feeling safe at home and having none or one person to count on in difficult times were common predictors of depressive and anxiety symptoms among Lebanese adults and Syrian refugees or migrants. Not having a legal residency permit was also a predictor of depressive symptoms for Syrian refugees or migrants. Prediction models developed for depressive and anxiety symptoms among both nationalities had good performance measures.

**Conclusions:**

The predictors of poor mental health included financial, health and social indicators for both Lebanese adults and Syrian refugees or migrants during the concurrent crisis in Lebanon. These models are most applicable in similar urban, crisis-affected and low-resource settings. Findings emphasise the need for social protection and financial support among populations with vulnerabilities.

**STRENGTHS AND LIMITATIONS OF THIS STUDY**

- The study sample is a probability sample of diverse populations living in low socio-economic status areas in Sin-El-Fil, Beirut, Lebanon, and is one of the few population-based studies on all aged adults in Lebanon.
- Replicable prediction models for depressive and anxiety symptoms were developed among Lebanese adults and Syrian refugees or migrants residing in Sin-El-Fil.
- This study identified predictors of poor mental health among vulnerable populations, which can inform service delivery and public health planning.

## Introduction

 Poor mental health, including depressive and anxiety symptoms, was a substantial contributor to the Global Burden of Disease before the COVID-19 pandemic.^1^ The WHO estimated that the COVID-19 pandemic elevated the prevalence of depression and anxiety by 25%.^2^ This decline in mental health was multifactorial and attributed to several stressors, including limited social interaction, fear of infection, loss of friends or family and lack of ‘normal’ routine. The pandemic also resulted in a loss of income for populations, particularly among those employed in informal sectors and individuals facing an increased care burden from homeschooling or elderly support.^3 4^ The distribution of these stressors was disparate between different populations; they also exacerbated health inequalities and disproportionately increased the prevalence of poor mental health among populations with low socio-economic status (SES)^5^ and women.^6^

Lebanon, a lower-middle income country in the Middle East and North Africa region, is an example of a context that possesses many stressors that impact well-being. Lebanon has been undergoing a protracted political and economic crisis since 2019, with severe devaluation of the local currency that led to a significant portion of the population moving below the poverty line.^7^ Additionally, Lebanon hosts the highest number of refugees per capita in the globe, where one quarter of the population are refugees, with an estimated 1.5 million from Syria.^8^ Unlike refugee populations in other contexts, many refugees and migrants in Lebanon live in residential areas alongside the Lebanese host population. Host populations with low SES, refugees and migrants, have stressors that place them at greater risk of poor mental health, including lack of employment opportunities, limited social mobility and lack of healthcare access.^9 10^ Refugees also have the added burden of family member loss from conflict, discrimination, fear of deportation and psychological trauma.^10 11^

Forced displacement is increasing globally due to the increase in conflict and climate-related events.^10 12^ Most studies on poor mental health have explored its risk either among host or refugee populations separately. In Lebanon, for example, studies identifying the predictors of poor mental health were restricted to older Syrian refugees, widowed Syrian refugee women and Syrian refugees who experienced loss of a close family member.^13^,^15^ In the context of Lebanon, there remains an opportunity to explore the predictors of poor mental health among both host and refugee populations with the aim of identifying the common and disparate predictors of poor mental health to inform service delivery and public health planning. Therefore, the aim of this study was to develop separate prediction models for depressive and anxiety symptoms among Lebanese adults and adult Syrian refugees or migrants residing in low SES areas of a suburb in Beirut, Lebanon.

## Methods

### Study design and setting

This study was a nested cross-sectional analysis from the first wave of a longitudinal study. The aim of the parent study was to explore the needs of subpopulations that were vulnerable to the increased risk of COVID-19 infection or its impact in Sin-El-Fil, a suburb east of Beirut, Lebanon.^16^ Participants in the parent study included: (1) adults living in low SES areas, (2) older adults (60 years or older), (3) adult Syrian refugees or migrants and (4) pregnant adult women.

### Sampling and study population

The study followed a multistage stratified sampling design using an area-based sampling method.^17^ The suburb of Sin-El-Fil was stratified into areas of high and low SES, where the boundaries were geographically defined based on stakeholder consultation with the Sin-El-Fil municipality and non-governmental organisations (NGOs). A household listing exercise was carried out whereby all households in Sin-El-Fil were enumerated face-to-face to complete an eligibility screening survey that determined age, nationality and pregnancy status. This identified households and individuals with the specific vulnerability criteria for inclusion in the parent study.^18^ Preconsent was obtained from all households with eligible individuals, and phone numbers were collected from consenting adults.

The parent study included participants with specific vulnerabilities from high and low SES areas. In high SES areas, all adult Syrian refugees or migrants, all pregnant adult women and all older adults were included. In low SES areas, all adult Syrian refugees or migrants and all pregnant adult women were included, while older adults and adults aged 18–60 years were randomly selected using proportionate allocation. The present study included only adult participants residing in low SES areas who completed the first wave of data collection conducted between June and October 2022 (n=2045), and the analysis was restricted to Lebanese adults and adult Syrian refugees or migrants (n=1986); individuals of any other nationality were excluded^19 20^ (online supplemental figure 1).

Respondents were contacted to complete a computer assisted telephone survey conducted by trained data collectors where data were entered into SurveyCTO software (Dobility, Cambridge, Massachusetts, USA). Verbal informed consent to participate in the telephone interview was obtained from selected respondents. Older adults had their ability to participate in the study assessed using five items altered from the University of California, San Diego, Brief Assessment of Capacity to Consent.^21^ Participants who scored a total below 7 were not eligible to participate (online supplemental table 1).

### Data sources

A survey was developed by integrating existing questionnaire modules, community-identified priorities, and adapting questions to the Sin-El-Fil context. The survey was developed in English and then translated to Arabic.^22^ The survey was created in partnership with representatives of the Sin-El-Fil municipality, the ministry of public health and NGOs operating in Sin-El-Fil. The survey was internally piloted to ensure the questions were correctly measuring what the study aimed to measure. Data were monitored weekly and call back checks were conducted to ensure accuracy of the collected data whereby a random set of questions were readministered to 5% of the sample for verification purposes.

### Outcome measures

Depressive and anxiety symptoms were the primary and secondary outcomes of interest in the study, respectively. Depressive symptoms were measured through the Patient Health Questionnaire-9 (PHQ-9). The PHQ-9 includes nine items assessing depressive symptoms and is validated in Arabic (online supplemental table 2). The total summative score of the PHQ-9 items ranges from 0 to 27 where each item is scored from 0 (not at all) to 3 (nearly every day).^23^ Anxiety symptoms were measured through the Generalised Anxiety Disorder-7 (GAD-7). The GAD-7 includes seven items assessing anxiety symptoms and is validated in Arabic (online supplemental table 3). The GAD-7 items’ total summative score ranges from 0 to 21 where each item is scored from 0 (not at all) to 3 (nearly every day).^24^ The PHQ-9 and GAD-7 had excellent reliability in this population (Cronbach’s alpha=0.89 and 0.95, respectively). A total summative score of 10 or more on the PHQ-9 and GAD-7 indicated the presence of depressive and anxiety symptoms, respectively, and was used as a cut-off point for the outcomes in this study.^23 24^

### Candidate predictors

Predictors of depressive and anxiety symptoms included for model development were identified based on the literature.^13^,^15^

To develop prediction models for depressive and anxiety symptoms among the Lebanese subpopulation, fourteen candidate predictors were included: sex, age, education, marital status, number of chronic illnesses, pain that impacts usual activity, household water insecurity, household food insecurity, employment status, debt status, perceived safety inside home, housing tenure status, household eviction notice and number of people to count on in difficult times.

Fifteen candidate predictors were included for the development of the depressive and anxiety symptoms prediction models among the Syrian subpopulation. These predictors were: sex, age, education, marital status, number of chronic illnesses, pain that impacts usual activity, household water insecurity, household food insecurity, employment status, debt status, perceived safety inside home, household eviction notice, number of people to count on in difficult times, receipt of cash assistance and legal residency permit possession characterised by having a government-issued document allowing Syrian refugees or migrants to legally reside in Lebanon.

### Statistical analysis

Both outcomes, using the PHQ-9 and GAD-7, were classified as binary (score <10 versus ≥10). Almost all candidate predictors were categorical except for age and number of chronic illnesses which were linearly associated with both outcomes. The study weights were calculated as the product of the baseline sampling weight (inverse probability of selection in the study sample) and the non-response adjustment factor. The non-response weight was estimated using a response propensity logistic regression model in which response status was modelled as a function of observed covariates to account for attrition during data collection. Further description of study weight computation is presented elsewhere.^20^ Sampling weights were used for descriptive estimates only. The number of Lebanese adults and Syrian refugees or migrants within each category of candidate predictors, along with their survey-weighted percentages, unadjusted weighted odds ratios (ORs) and 95% confidence intervals (CIs) were calculated (tables1  2 and online supplemental tables 4,5). Estimates with p-values less than 0.05 were considered statistically significant.

**Table 1:** Characteristics of Lebanese participants and their association with depressive symptoms

	Total		PHQ-9 score <10		PHQ-9 score ≥10*		OR	(95% CI)
	n=1290	(100%)	n=966	(74.7%)	n=324	(25.3%)		
Age, median (IQR)	50	(35–63)	50	(33–63)	53	(39–63)	1.01	(1.00 to 1.01)
Missing	8		8		0			
Sex								
Male	584	(45.5)	439	(75.2)	145	(24.8)	1.00	
Female	706	(54.5)	527	(74.4)	179	(25.6)	1.05	(0.81 to 1.35)
Education								
School not attended	98	(8.4)	53	(54.1)	45	(45.9)	1.00	
School not completed	574	(47.6)	407	(70.9)	167	(29.1)	0.49	(0.31 to 0.75)
School completed	174	(14.1)	146	(83.3)	28	(16.7)	0.24	(0.13 to 0.42)
Vocational	115	(9.3)	85	(74.7)	30	(25.3)	0.40	(0.22 to 0.72)
Higher education	252	(20.6)	208	(82.9)	44	(17.1)	0.24	(0.15 to 0.41)
Missing	77		67		10			
Marital status								
Single/engaged	435	(34.0)	349	(80.1)	86	(19.9)	1.00	
Married	710	(54.4)	519	(73.0)	191	(27.0)	1.49	(1.11 to 1.99)
Divorced/separated/widowed	145	(11.6)	98	(67.4)	47	(32.6)	1.94	(1.27 to 2.97)
Pain that impacts usual activity								
No	926	(71.5)	722	(78.1)	204	(21.9)	1.00	
Yes	361	(28.5)	241	(66.2)	120	(33.8)	1.81	(1.38 to 2.38)
Missing	3		3		0			
Number of chronic illnesses, range (0–6), median (IQR)	0	(0–1)	0	(0–1)	0	(0–1)	1.18	(1.05 to 1.32)
Eviction notice								
No	616	(47.3)	451	(73.0)	165	(27.0)	1.00	
Yes	79	(5.9)	49	(61.0)	30	(39.0)	1.72	(1.05 to 2.83)
Owned	584	(46.8)	457	(78.2)	127	(21.8)	0.75	(0.58 to 0.98)
Missing	11		9		2			
Household water insecurity								
Secure	374	(30.1)	315	(84.2)	59	(15.8)	1.00	
Insecure	876	(69.9)	615	(70.1)	261	(29.9)	2.27	(1.65 to 3.12)
Missing	40		36		4			
Household food insecurity								
Secure	419	(35.0)	388	(92.7)	31	(7.3)	1.00	
Insecure	795	(65.0)	529	(66.2)	266	(33.8)	6.46	(4.33 to 9.64)
Missing	76		49		27			
Employment status								
No	713	(56.2)	518	(72.5)	195	(27.5)	1.00	
Yes	570	(43.8)	443	(77.7)	127	(22.3)	0.76	(0.58 to 0.98)
Missing	7		5		2			
Debt								
No	853	(73.4)	693	(80.8)	160	(19.2)	1.00	
Yes	321	(26.6)	180	(56.0)	141	(44.0)	3.31	(2.49 to 4.39)
Missing	116		93		23			
Perceived safety at home								
Very safe	729	(57.3)	581	(79.6)	148	(20.4)	1.00	
Somewhat safe	506	(39.6)	355	(70.2)	151	(29.8)	1.66	(1.27 to 2.16)
Not safe at all	40	(3.1)	21	(51.4)	19	(48.6)	3.68	(1.92 to 7.05)
Missing	15		9		6			
Housing tenure status								
Rented	623	(48.0)	440	(70.2)	183	(29.8)	1.00	
Owned	633	(50.9)	501	(79.0)	132	(21.0)	0.63	(0.48 to 0.81)
Other/hosted	13	(1.1)	11	(83.8)	2	(16.2)	0.46	(0.10 to 2.09)
Missing	21		14		7			
Number of people to count on in difficult times								
None	223	(17.2)	144	(63.4)	79	(36.6)	1.00	
One	447	(35.2)	306	(68.5)	141	(31.5)	0.80	(0.57 to 1.13)
Two to five	580	(45.0)	485	(83.8)	95	(16.2)	0.34	(0.23 to 0.48)
Six and more	32	(2.6)	24	(75.5)	8	(24.5)	0.56	(0.24 to 1.32)
Missing	8		7		1			

* PHQ-9 score ≥10 indicates having depressive symptoms.

PHQ-9, Patient Health Questionnaire-9.

**Table 2:** Characteristics of Syrian participants and their association with depressive symptoms

	Total		PHQ-9 score <10		PHQ-9 score ≥10*		OR	(95% CI)
	n=657	(100%)	n=368	(56.1%)	n=289	(43.9%)		
Age, median (IQR)	34	(26–41)	34	(24–40)	34	(26–43)	1.01	(1.00 to 1.03)
Missing	2		0		2			
Sex								
Male	334	(50.1)	191	(57.2)	143	(42.8)	1.00	
Female	323	(49.9)	177	(55.1)	146	(44.9)	1.09	(0.78 to 1.51)
Education								
School not attended	95	(15.2)	34	(39.4)	61	(60.6)	1.00	
School not completed	444	(70.5)	261	(57.7)	183	(42.3)	0.48	(0.29 to 0.78)
School completed	51	(8.5)	33	(68.7)	18	(31.3)	0.30	(0.14 to 0.63)
Vocational	18	(2.9)	9	(56.0)	9	(44.0)	0.51	(0.18 to 1.49)
Higher education	18	(2.9)	12	(65.3)	6	(34.7)	0.35	(0.11 to 1.09)
Missing	31		19		12			
Marital status								
Single/engaged	107	(16.4)	72	(66.1)	35	(33.9)	1.00	
Married	518	(78.5)	282	(55.1)	236	(44.9)	1.59	(0.99 to 2.54)
Divorced/separated/widowed	32	(5.1)	14	(39.3)	18	(60.7)	3.01	(1.27 to 7.15)
Pain that impacts usual activity								
No	441	(67.0)	275	(61.9)	166	(38.1)	1.00	
Yes	216	(33.0)	93	(44.3)	123	(55.7)	2.05	(1.43 to 2.92)
Number of chronic illnesses, range (0–4), median (IQR)	0	(0–0)	0	(0–0)	0	(0–1)	1.34	(1.02 to 1.77)
Eviction notice								
No	489	(75.0)	300	(60.9)	189	(39.1)	1.00	
Yes	163	(25.0)	66	(42.3)	97	(57.7)	2.12	(1.44 to 3.13)
Missing	5		2		3			
Household water insecurity								
Secure	157	(25.1)	101	(64.6)	56	(35.4)	1.00	
Insecure	474	(74.9)	242	(51.2)	232	(48.8)	1.74	(1.17 to 2.60)
Missing	26		25		1			
Household food insecurity								
Secure	60	(10.2)	53	(88.3)	7	(11.7)	1.00	
Insecure	549	(89.8)	288	(52.6)	261	(47.4)	6.77	(2.83 to 16.21)
Missing	48		27		21			
Employment status								
No	392	(61.1)	209	(53.8)	183	(46.2)	1.00	
Yes	265	(38.9)	159	(59.8)	106	(40.2)	0.78	(0.56 to 1.09)
Cash assistance								
No	351	(56.0)	205	(59.2)	146	(40.8)	1.00	
Yes	283	(44.0)	154	(54.0)	129	(46.0)	1.24	(0.88 to 1.73)
Missing	23		9		14			
Debt								
No	135	(23.0)	109	(78.5)	26	(21.5)	1.00	
Yes	478	(77.0)	238	(49.9)	240	(50.1)	3.66	(2.21 to 6.06)
Missing	44		21		23			
Perceived safety at home								
Very safe	367	(54.8)	240	(65.0)	127	(35.0)	1.00	
Somewhat safe	242	(38.9)	117	(49.8)	125	(50.2)	1.87	(1.31 to 2.67)
Not safe at all	45	(6.3)	10	(20.6)	35	(79.4)	7.17	(3.39 to 15.14)
Missing	3		1		2			
Number of people to count on in difficult times								
None	158	(24.3)	100	(63.3)	58	(36.7)	1.00	
One	257	(38.8)	94	(38.2)	163	(61.8)	2.79	(1.80 to 4.33)
Two to five	227	(34.9)	164	(69.9)	63	(30.1)	0.74	(0.47 to 1.19)
Six and more	12	(2.0)	9	(80.5)	3	(19.5)	0.42	(0.11 to 1.67)
Missing	3		1		2			
Legal residency permit								
No	517	(79.1)	274	(52.9)	243	(47.1)	1.00	
Yes	131	(20.9)	89	(69.7)	42	(30.3)	0.49	(0.32 to 0.75)
Missing	9		5		4			

* PHQ-9 score ≥10 indicates having depressive symptoms.

PHQ-9, Patient Health Questionnaire-9.

Adaptive Least Absolute Shrinkage and Selection Operator (LASSO) logistic regression models were implemented to identify the predictors of depressive and anxiety symptoms among Lebanese adults and Syrian refugees or migrants. For prediction, an initial standard penalised regression model was fitted to obtain preliminary coefficient estimates, which were then used to construct variable-specific penalty weights. A penalty-weighted LASSO model was subsequently refitted, allowing differential shrinkage across predictors. The penalty strength (λ) was selected using 10-fold cross-validation, where the dataset was partitioned into 10 folds, with models iteratively trained on nine folds and evaluated on the remaining fold.^25^ The λ value which minimised the mean cross-validated prediction error, was retained for the final model. The penalised models represented the primary analysis. Further information on the prediction models is presented in the Supplemental Appendix (online supplemental tables 6,7). Separate adjusted logistic regressions were run on selected predictors to extract ORs and 95% CIs for coefficient interpretability.

The final models’ performances were assessed through their discriminative abilities using the area under the receiver operating characteristics curve (AUC). A model’s AUC ranges from 0.5 to 1 with an AUC of 1 indicating perfect discrimination between distinct outcome classes. Additionally, the calibration slope (C-Slope) and calibration plots were generated to assess model calibration. A model with perfect calibration has a slope of 1, indicating a perfect match between predicted and observed values.^26^ The calibration-in-the-large (CITL) was computed to compare the observed number of events to the average predictive risk. The expected-to-observed ratio (E:O) was calculated as the ratio of the expected number of events (sum of predicted probabilities) to the observed number of events. The pattern of missingness was tested and presented in the methods section of the online supplemental appendix 1.^27^ Missing values were missing at random and a complete-case analysis was implemented. Further information on the impact of missing data and complete-case analysis is presented in the online supplemental appendix 1.

Transparent Reporting of a Multivariable Prediction Model for Individual Prognosis or Diagnosis (TRIPOD) and Strengthening the Reporting of Observational Studies in Epidemiology (STROBE) guidelines were used to inform the reporting of this manuscript.^28 29^

### Patient and public involvement

Patients and/or the public were not involved in the design, or conduct, or reporting, or dissemination plans of this research.

## Results

A total of 2045 respondents participated in this study. The study population after restriction to Lebanese and Syrian nationalities included 1986 participants of which 1322 (66.5%) were Lebanese adults and 664 (33.5%) were Syrian refugees or migrants (online supplemental figure 1). Among Lebanese participants, 324 (25.3%) were classified as having depressive symptoms and 402 (30.9%) were classified as having anxiety symptoms. The median age for Lebanese participants was 50 (IQR: 35–63) years, and 45.5% were males (table 1 and online supplemental table 4).

The median age for Syrian participants was 34 (IQR: 26–41) years, of which 50.0% were males. From those participants, 289 (43.9%) were classified as having depressive symptoms, and 321 (47.2%) were classified as having anxiety symptoms (table 2 and online supplemental table 5). Interpretations of the unadjusted ORs and 95%CIs are presented in the online supplemental appendix 1.

**Figure 1:**
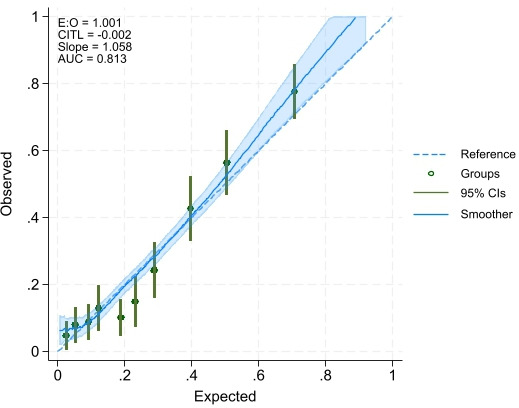
Calibration plot of depressive symptoms prediction model among Lebanese adults. AUC, area under the receiver operating characteristics curve; CITL, calibration-in-the-large; E:O, expected-to-observed ratio.

The model predicting depressive symptoms among Lebanese adults retained ten predictors: education, number of chronic illnesses, pain that impacts usual activity, household eviction notice, household water insecurity, household food insecurity, debt status, perceived safety inside home, housing tenure status and number of people to count on in difficult times. This model had an AUC=0.81; 95% CI 0.78 to 0.84, C-Slope=1.06; 95% CI 0.90 to 1.21, a CITL of −0.002 and E:O of 1.001 (figure 1 and online supplemental table 6). Predictors from the model predicting anxiety symptoms among Lebanese adults are presented in online supplemental table 6. Similarly, the model had an AUC=0.80; 95% CI 0.77 to 0.83, C-Slope=1.02; 95% CI 0.87 to 1.17, a CITL of 0.004 and E:O of 0.998 (online supplemental figure 2, table 6).

**Figure 2:**
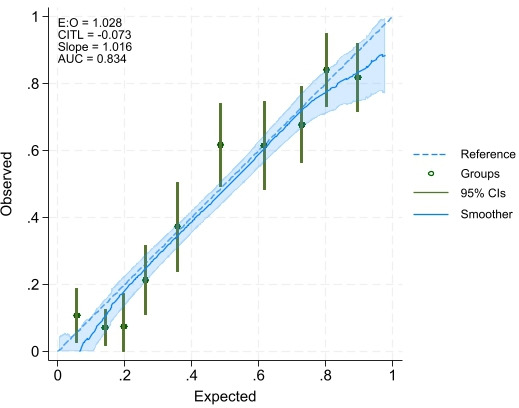
Calibration plot of depressive symptoms prediction model among Syrian refugees or migrants. AUC, area under the receiver operating characteristics curve; CITL, calibration-in-the-large; E:O, expected-to-observed ratio.

The model predicting depressive symptoms among Syrian refugees or migrants retained ten predictors: education, marital status, number of chronic illnesses, pain that impacts usual activity, household eviction notice, household food insecurity, debt status, perceived safety inside home, number of people to count on in difficult times and legal residency permit possession. This model had an AUC=0.83; 95% CI 0.80 to 0.88, C-Slope=1.02; 95% CI 0.84 to 1.19, a CITL of −0.073 and E:O of 1.028 (figure 2 and online supplemental table 7). Predictors of the model predicting anxiety symptoms among Syrian refugees or migrants are presented in online supplemental table 7. Similarly, the model had an AUC=0.72; 95% CI 0.68 to 0.76, C-Slope=1.01; 95% CI 0.79 to 1.23, a CITL of −0.031 and E:O of 1.014 (online supplemental figure 3, table 7).

Having pain that impacts usual activity, having debt, not feeling safe at home and having none or one person to count on in difficult times were common predictors of depressive and anxiety symptoms among Lebanese adults and Syrian refugees or migrants. Additional details on the predictors of anxiety symptoms along with their model evaluation metrics among both populations are presented in the results section of the online supplemental appendix 1.

## Discussion

This study explored predictors of depressive and anxiety symptoms among Lebanese adults and Syrian refugees or migrants residing in low SES areas of Sin-El-Fil. Four prediction models were developed, and their discrimination and calibration were assessed. These models identified common and disparate stressors of depressive and anxiety symptoms among Lebanese adults and Syrian refugees or migrants residing in Sin-El-Fil. The findings of this study also showed a high prevalence of depressive and anxiety symptoms, and several predictors of poor mental health were common between both populations residing in low SES areas in Sin-El-Fil.

Aligned with a multicountry representative study, which examined the prevalence of depressive symptoms among refugees and their host population in Kenya, Uganda and Ethiopia, the prevalence of depressive symptoms among refugees and migrants was also higher than that of the host population living in similar contexts.^30^ Moreover, the overall prevalence of depressive symptoms among Syrian refugees or migrants in this study was higher than the pooled estimate of depressive symptoms among refugees and migrants reported in a global systematic review of 81 studies and 53 458 participants (26.4%; 95% CI 22.2% to 31.1%).^31^

Similar to other prediction models for poor mental health among Syrian refugees or migrants, this study found that having at least one chronic illness, being in debt, receiving a household eviction notice, having pain that impacts usual activity, living in a food insecure household and not having a legal residency permit were predictors of depressive symptoms.^13 14^ Additional predictors not previously reported among Syrian refugees or migrants in Lebanon included not attending school, not feeling safe at all at home and having limited social support.

Similar to other studies, financial predictors of poor mental health among both Lebanese and Syrian populations included being in debt, living in a food insecure household and receiving an eviction notice.^13^,^15^ Financial stressors contribute to poor mental health through the inability to meet one’s basic needs and overall deteriorating living conditions.^1032^,^34^ The economic crisis, including the devaluation of currency, loss of savings and lack of employment opportunities in Lebanon has pushed many households into poverty.^35^ As a result, many households were unable to meet their basic needs, including their ability to pay rent and obtain enough food, which has forced many households into debt.^36^

Consistent with findings from other studies, being widowed or divorced and having no one to count on during the protracted crisis can intensify loneliness and impact mental health.^37^,^39^ This compounded isolation often makes it difficult to cope with crisis stressors.^37^,^39^ Thus, with the lack of strong social support networks and prolonged economic crisis, the risk of developing depressive symptoms increases.^39 40^

Aligned with a longitudinal study among French participants, not feeling safe at home was a predictor of depressive symptoms among Lebanese adults and Syrian refugees or migrants.^41^ In previous studies, the feeling of safety at home was mainly measured through domestic and intimate partner violence.^41^,^43^ These studies reported increased levels of domestic and intimate partner violence and isolation during the COVID-19 pandemic.^41^,^43^ Exposure to violence at home has contributed to developing poor mental health including depressive symptoms.^41 44^

Furthermore, the lack of a legal residency permit was a predictor of depressive symptoms among Syrian refugees or migrants in Lebanon, and this was comparable with another study.^13^ Findings have suggested that being undocumented results in migrants and refugees experiencing economic uncertainty, being unable to access essential services such as healthcare services and being at risk of deportation.^19 45^ Therefore, it remains essential to remove the financial and administrative barriers for migrants to obtain and renew their legal documentation.

The inclusion of structural variables, such as the possession of a legal residency permit, captures broader determinants of vulnerability. These models require careful ethical considerations to minimise stigma and avoid unintended harm to the vulnerable populations they include. Furthermore, such models should be used exclusively for supportive outreach, resource allocation and service prioritisation. Robust governance frameworks, transparency in application and safeguards against discriminatory use are essential.

This study had limitations and strengths. The model was developed using data from low SES areas in Sin-El-Fil, Lebanon, recruited through a telephone survey. Thus, findings and model performance are context-specific and may not be generalisable to all Lebanese and Syrian populations in Lebanon. External validation among independent Lebanese and Syrian populations is required before wider use of the developed models. On the other hand, this study sample is representative of individuals living in low SES areas in Sin-El-Fil. While the PHQ-9 and GAD-7 have been rigorously validated in Arabic for face-to-face and self-report formats, specific psychometric validation of their Arabic versions when administered via telephone remains limited. The measurement invariance was not formally tested across nationality. However, both groups share Arabic as a common language and substantial cultural and social overlap within the same geographical context, which may reduce the likelihood of major construct-level differences.

Within the broader conflict-migration mental health literature, there is increasing recognition that resource-constrained humanitarian settings require pragmatic, scalable approaches to identify individuals at highest risk.^10 13 14^ Developing and evaluating prediction models for depressive and anxiety symptoms using the PHQ-9 and GAD-7 may support targeted screening and triage strategies, enabling more efficient allocation of limited mental health resources.^10 13 14^ The models help identify individuals at a higher current probability of screening positive for having depressive and anxiety symptoms and support targeted screening and service planning. However, the retained predictors should not be interpreted as causal.

In conclusion, this study sheds light on the widespread presence of depressive and anxiety symptoms among adults residing in low SES areas of Sin-El-Fil. It highlights that Syrian refugees or migrants and their host population in Lebanon have similar predictors of depressive and anxiety symptoms, which are mainly related to SES characteristics, physical health and social isolation. The findings of this study provide insight into understanding the risk factors of mental health disorders, offering a basis for targeting interventions to high-risk populations to reduce the associated burden in Lebanon.

## Supplementary material

10.1136/bmjopen-2025-101258 online supplemental table 1 1

## Data Availability

The anonymised data can be obtained upon reasonable request from the Center for Research on Population and Health at the American University of Beirut (crph@aub.edu.lb).
