# Supplementary material for: Predicting depressive and anxiety symptoms among Lebanese and Syrian adults in a suburb of Beirut, Lebanon, during concurrent crises: nested cross-sectional study
Source: BMJ Open. 2026 May 11;16(5):e101258. doi: 10.1136/bmjopen-2025-101258 (PMC13182334; doi:10.1136/bmjopen-2025-101258)

Supplemental Appendix

Predicting depressive and anxiety symptoms among Lebanese and Syrian adults in a suburb of Beirut, Lebanon, during concurrent crises: nested cross-sectional study

Authors

Hazar Shamas MSc[1], Marie-Elizabeth Ragi MSc[1], Berthe Abi Zeid MPH[1], Jocelyn DeJong PhD[2], Stephen J. McCall DPhil[1]*, CAEP Study Group[3]¶

Affiliations

[1]Center for Research on Population and Health, Faculty of Health Sciences, American University of Beirut, Beirut, Lebanon

[2]Department of Epidemiology and Population Health, Faculty of Health Sciences, American University of Beirut, Beirut, Lebanon

[3]Faculty of Health Sciences, American University of Beirut, Beirut, Lebanon

*Corresponding author: Stephen J. McCall, Center for Research on Population and Health, Faculty of Health Sciences, American University of Beirut, Beirut 1107 2020, Lebanon; sm227@aub.edu.lb

Members of CAEP study group:

Aline Germani, Fadi El-Jardali, Hala Ghattas, Nada M. Melhem, Jocelyn DeJong, and Stephen J. McCall

Contents

METHODS ... 3

Figure S1. Flow diagram representing the participants from low SES areas of Sin-El-Fil included in the study ... 3

Table S1. University of California, San Diego, Brief Assessment of Capacity to Consent (UBACC) items ... 4

Table S2. Patient Health Questionnaire-9 (PHQ-9) ... 4

Table S3. Generalised Anxiety Disorder 7-item (GAD-7) scale ... 5

RESULTS ... 6

Table S4. Characteristics of Lebanese participants and their association with anxiety symptoms ... 8

Table S5. Characteristics of Syrian participants and their association with anxiety symptoms ... 10

Table S6. Predictors of depressive and anxiety symptoms among Lebanese adults ... 12

Table S7. Predictors of depressive and anxiety symptoms among Syrian refugees or migrants ... 14

Figure S2. Calibration plot of anxiety symptoms prediction model among Lebanese adults ... 16

Figure S3. Calibration plot of anxiety symptoms prediction model among Syrian refugees or migrants ... 17

METHODS

Figure S1. Flow diagram representing the participants from low SES areas of Sin-El-Fil included in the study

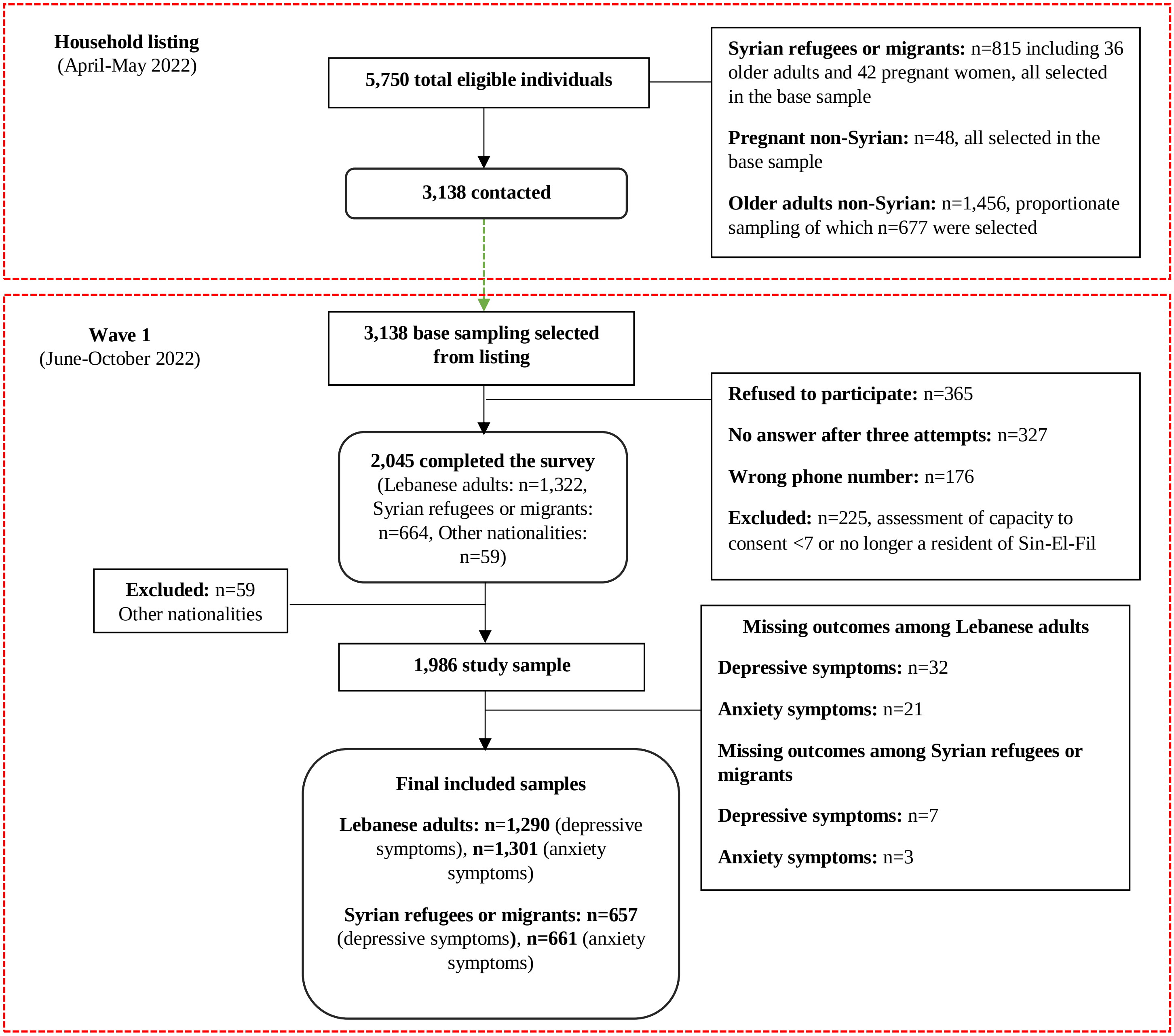

Assessment of capacity to consent

Table S1. University of California, San Diego, Brief Assessment of Capacity to Consent (UBACC) items

1.What is the purpose of the study that was just described to you? **(2 = the objective of the study)**	0	1	2
2.Do you believe this is primarily research or primarily treatment? **(2 = Research)**	0	1	2
3.Do you have to be in this study if you do not want to participate? **(2 = No)**	0	1	2
4.If you participate in this study, what are some of the things that you will be asked to do? **(2= Answer questions)**	0	1	2
5.Is it possible that being in this study will not have any benefit to you? **(2 = Yes)**	0	1	2

Patient Health Questionnaire-9 (PHQ-9) items

Table S2. Patient Health Questionnaire-9 (PHQ-9)

Over the last 2 weeks, how often have you been bothered by any of the following problems?	Not at all	Several days	More than half of the days	Nearly everyday
1.Little interest or pleasure in doing things	0	1	2	3
2.Feeling down, depressed, or hopeless	0	1	2	3
3.Trouble falling or staying asleep or sleeping too much	0	1	2	3
4.Feeling tired or having little energy	0	1	2	3
5.Poor appetite or overeating	0	1	2	3
6.Feeling bad about yourself – or that you are a failure or have let yourself or your family down	0	1	2	3
7.Trouble concentrating on things, such as reading the newspaper or watching television	0	1	2	3
8.Moving or speaking so slowly that other people could have noticed? Or the opposite -being so fidgety or restless that you have been moving around a lot more than usual	0	1	2	3
9.Thoughts that you would be better off dead or of hurting yourself in some way	0	1	2	3

Generalised Anxiety Disorder 7-item scale items

Table S3. Generalised Anxiety Disorder 7-item (GAD-7) scale

Over the last 2 weeks, how often have you been bothered by any of the following problems?	Not at all	Several days	More than half of the days	Nearly everyday
1.Feeling nervous, anxious, or on edge	0	1	2	3
2.Not being able to stop or control worrying	0	1	2	3
3.Worrying too much about different things	0	1	2	3
4.Trouble relaxing	0	1	2	3
5.Being so restless that it is hard to sit still	0	1	2	3
6.Becoming easily annoyed or irritable	0	1	2	3
7.Feeling afraid, as if something awful might happen	0	1	2	3

Analysis of missingness pattern

To explore the pattern of missingness among candidate predictors only, an indicator variable for each existing candidate predictor with missing data was generated. A value of 1 was given for every missing observation and a value of 0 was given if an observation was not missing. Logit models were then run to test if any of the candidate predictors could predict whether a given observation was missing [1].

Impact of missing data and complete-case analysis

The exclusion of observations with missing data did not alter the effective sample size or the events-per-parameter ratio.

References:

1- University of Wisconsin-Madison. Multiple Imputation in Stata: Deciding to Impute 2013 [online]. 13 Jan 2013. https://sscc.wisc.edu/sscc/pubs/stata_mi_decide.htm (accessed 7 Jul 2024).

RESULTS

Significant unadjusted ORs representing association between depressive and anxiety symptoms among Lebanese adults and Syrian refugees or migrants along with their 95%CIs:

Among the Lebanese participants, some protective factors against depressive symptoms were completing higher education compared to not attending school (OR:0.24; 95%CI: 0.15-0.41) and being employed compared to not being employed (OR:0.76; 95%CI: 0.58-0.98). Factors associated with having depressive symptoms included: being married and being divorced/ separated/ widowed compared to being single (OR:1.49; 95%CI: 1.11-1.99) and (OR:1.94; 95%CI: 1.27-2.97) respectively, having pain that impacts usual activity compared to none (OR:1.81; 95%CI: 1.38-2.38), receiving an eviction notice compared to none (OR:1.72; 95%CI: 1.05-2.83), living in a food and water insecure household compared to living in a food and water secure household (OR:6.46; 95%CI: 4.33-9.64) and (OR:2.27; 95%CI: 1.65-3.12) respectively, being in debt compared to not having debt (OR:3.31; 95%CI: 2.49-4.39), and not feeling safe at all compared to feeling very safe at home (OR:3.68; 95%CI: 1.92-7.05) (Table 1).

Factors protective against anxiety symptoms were completing higher education compared to not attending school (OR:0.27; 95%CI: 0.16-0.44), living in their owned home compared to living in a rented home (OR:0.75; 95%CI: 0.59-0.95), having two to five or six and more people to count on in difficult times compared to having none (OR:0.40; 95%CI: 0.29-0.56) and (OR:0.37; 95%CI: 0.15-0.90) respectively. Factors associated with having anxiety symptoms included: having pain that impacts usual activity compared to none (OR:1.52; 95%CI: 1.18-1.97), receiving an eviction notice compared to none (OR:1.83; 95%CI: 1.13-2.97), living in a food insecure household compared to living in a food secure household (OR:4.07; 95%CI: 2.97-5.60), being in debt compared to not having debt (OR:3.97; 95%CI: 3.01-5.24), not feeling safe at all compared to feeling very safe at home (OR:3.24; 95%CI: 1.69-6.18) (Supplemental Table 4).

Among the Syrian participants, factors protective against developing depressive symptoms were completing school compared to not attending school (OR:0.30; 95%CI: 0.14-0.63) and possessing a legal residency permit compared to none (OR:0.49; 95%CI: 0.32-0.75). Factors associated with developing depressive symptoms included having pain that impacts usual activity compared to none (OR:2.05; 95%CI: 1.43-2.92), receiving an eviction notice compared to none (OR:2.12; 95%CI: 1.44-3.13), living in a food and water insecure household compared to living in a food and water secure household (OR:6.77; 95%CI: 2.83-16.21) and (OR:1.74; 95%CI: 1.17-2.60) respectively, being in debt compared to not having debt (OR:3.66; 95%CI: 2.21-6.06), and not feeling safe at all compared to feeling very safe at home (OR:7.17; 95%CI: 3.39-15.14) (Table 2).

Factors protective against anxiety symptoms were completing higher education compared to not attending school (OR:0.31; 95%CI: 0.10-0.92), and having six or more people to count on in difficult times compared to none (OR:0.20; 95%CI: 0.04-0.99). Factors associated with developing anxiety symptoms were being married or divorced/ separated/ widowed compared to being single (OR:1.87; 95%CI: 1.18-2.96) and (OR:2.42; 95%CI: 1.01-5.81) respectively, having pain that impacts usual activity compared to none (OR:1.67; 95%CI: 1.17-2.37), receiving an eviction notice compared to none (OR:1.66; 95%CI: 1.13-2.43), living in a food and water insecure household compared to living in a food and water secure household (OR:3.68; 95%CI: 1.90-7.14) and (OR:1.49; 95%CI: 1.01-2.20) respectively, being in debt compared to not having

debt (OR:3.17; 95%CI: 1.99-5.04), and not feeling safe at all compared to feeling very safe at home (OR:3.39; 95%CI: 1.62-7.06) (Supplemental Table 5).

Table S4. Characteristics of Lebanese participants and their association with anxiety symptoms

	Total		GAD-7 score <10		GAD-7 score ≥10*		OR	(95%CI)
	n=1,301	(100%)	n=899	(69.1%)	n=402	(30.9%)		
**Age Median (IQR)**	50	(35-63)	50	(34-63)	51	(36-63)	1.00	(1.00-1.01)
Missing	8		6		2			
**Sex**								
Male	591	(45.6)	409	(69.2)	182	(30.8)	1.00	
Female	710	(54.4)	490	(69.1)	220	(30.9)	1.00	(0.79-1.27)
**Education**								
School not attended	101	(8.6)	52	(51.9)	49	(48.1)	1.00	
School not completed	578	(47.6)	387	(67.1)	191	(32.9)	0.53	(0.34-0.81)
School completed	175	(14.1)	131	(75.2)	44	(24.8)	0.35	(0.21-0.60)
Vocational	116	(9.3)	83	(72.1)	33	(27.9)	0.42	(0.24-0.74)
Higher education	252	(20.4)	202	(80.1)	50	(19.9)	0.27	(0.16-0.44)
Missing	79		44		35			
**Marital status**								
Single/ Engaged	437	(33.9)	316	(71.9)	121	(28.1)	1.00	
Married	718	(54.6)	491	(68.5)	227	(31.5)	1.18	(0.91-1.54)
Divorced/ Separated/ Widowed	146	(11.6)	92	(64.1)	54	(35.9)	1.44	(0.96-2.14)
**Pain that impacts usual activity**								
No	929	(71.1)	666	(71.7)	263	(28.3)	1.00	
Yes	370	(28.9)	231	(62.5)	139	(37.5)	1.52	(1.18-1.97)
Missing	2		2		0			
**Number of chronic illnesses Range (0-6) Median (IQR)**	0	(0-1)	0	(0-1)	0	(0-1)	1.09	(0.98-1.22)
**Eviction notice**								
No	623	(47.5)	431	(69.0)	192	(31.0)	1.00	
Yes	80	(5.9)	45	(54.9)	35	(45.1)	1.83	(1.13-2.97)
Owned	588	(46.7)	416	(71.0)	172	(29.0)	0.91	(0.71-1.17)
Missing	10		7		3			
**Household water insecurity**								
Secure	380	(30.3)	269	(70.8)	111	(29.2)	1.00	
Insecure	881	(69.7)	594	(67.5)	287	(32.5)	1.17	(0.90-1.53)
Missing	40		36		4			
**Household food insecurity**								
Secure	425	(35.3)	369	(86.8)	56	(13.2)	1.00	
Insecure	799	(64.7)	493	(61.6)	306	(38.4)	4.07	(2.97-5.60)
Missing	77		37		40			
**Employment status**								
No	720	(56.2)	484	(67.3)	236	(32.7)	1.00	
Yes	575	(43.8)	413	(71.9)	162	(28.1)	0.81	(0.63-1.03)

Missing	6		2		4			
**Debt**								
No	858	(73.4)	677	(78.7)	181	(21.3)	1.00	
Yes	324	(26.6)	155	(48.2)	169	(51.8)	3.97	(3.01-5.24)
Missing	119		67		52			
**Perceived safety at home**								
Very safe	731	(57.0)	553	(75.7)	178	(24.3)	1.00	
Somewhat safe	514	(39.9)	316	(61.5)	198	(38.5)	1.96	(1.53-2.51)
Not safe at all	40	(3.1)	20	(49.1)	20	(50.9)	3.24	(1.69-6.18)
Missing	16		10		6			
**Housing tenure status**								
Rented	629	(48.1)	416	(65.8)	213	(34.2)	1.00	
Owned	638	(50.9)	458	(72.0)	180	(28.0)	0.75	(0.59-0.95)
Other	13	(1.0)	10	(77.3)	3	(22.7)	0.57	(0.15-2.09)
Missing	21		15		6			
**Number of people to count on in difficult times**								
None	227	(17.2)	134	(58.5)	93	(41.5)	1.00	
One	451	(35.3)	277	(61.9)	174	(38.1)	0.87	(0.63-1.21)
Two to five	583	(44.8)	455	(77.9)	128	(22.1)	0.40	(0.29-0.56)
Six and more	32	(2.7)	25	(79.1)	7	(20.9)	0.37	(0.15-0.90)
Missing	8		8		0			

CI, confidence interval; OR, odds ratio; GAD-7, Generalised Anxiety Disorder-7

*GAD-7 score ≥10 indicates having anxiety symptoms.

Table S5. Characteristics of Syrian participants and their association with anxiety symptoms

	Total		GAD-7 score <10		GAD-7 score ≥10*		OR	(95%CI)
	n=661	(100%)	n=340	(52.8%)	n=321	(47.2%)		
**Age Median (IQR)**	34	(26-41)	34	(25-41)	34	(26-42)	1.00	(0.99-1.02)
Missing	2		0		2			
**Sex**								
Male	335	(49.9)	174	(52.8)	161	(47.2)	1.00	
Female	326	(50.1)	166	(52.8)	160	(47.2)	1.00	(0.72-1.39)
**Education**								
School not attended	95	(15.1)	38	(43.4)	57	(56.6)	1.00	
School not completed	448	(70.7)	235	(52.9)	213	(47.1)	0.68	(0.42-1.11)
School completed	51	(8.4)	31	(63.4)	20	(36.6)	0.44	(0.21-0.94)
Vocational	18	(2.9)	8	(51.2)	10	(48.8)	0.73	(0.25-2.14)
Higher education	18	(2.9)	12	(71.5)	6	(28.5)	0.31	(0.10-0.92)
Missing	31		16		15			
**Marital status**								
Single/ Engaged	107	(16.3)	69	(65.8)	38	(34.2)	1.00	
Married	522	(78.7)	258	(50.7)	264	(49.3)	1.87	(1.18-2.96)
Divorced/ Separated/ Widowed	32	(5.1)	13	(44.3)	19	(55.7)	2.42	(1.01-5.81)
**Pain that impacts usual activity**								
No	443	(66.9)	249	(57.1)	194	(42.9)	1.00	
Yes	218	(33.1)	91	(44.3)	127	(55.7)	1.67	(1.17-2.37)
**Number of chronic illnesses Range (0-4) Median (IQR)**	0	(0-0)	0	(0-0)	0	(0-0)	0.96	(0.75-1.22)
**Eviction notice**								
No	492	(75.0)	271	(56.1)	221	(43.9)	1.00	
Yes	164	(25.0)	67	(43.5)	97	(56.5)	1.66	(1.13-2.43)
Missing	5		2		3			
**Household water insecurity**								
Secure	157	(25.0)	90	(58.7)	67	(41.3)	1.00	
Insecure	478	(75.0)	226	(48.8)	252	(51.2)	1.49	(1.01-2.20)
Missing	26		24		2			
**Household food insecurity**								
Secure	60	(10.2)	46	(78.8)	14	(21.2)	1.00	
Insecure	553	(89.8)	272	(50.2)	281	(49.8)	3.68	(1.90-7.14)
Missing	48		22		26			
**Employment status**								
No	396	(61.3)	200	(52.8)	196	(47.2)	1.00	
Yes	265	(38.7)	140	(52.9)	125	(47.1)	1.00	(0.72-1.39)
**Cash assistance**								
No	355	(56.3)	197	(57.0)	158	(43.0)	1.00	
Yes	283	(43.7)	135	(48.7)	148	(51.3)	1.40	(1.00-1.95)
Missing	23		8		15			

**Debt**								
No	135	(23.0)	102	(75.3)	33	(24.7)	1.00	
Yes	482	(77.0)	228	(49.1)	254	(50.9)	3.17	(1.99-5.04)
Missing	44		10		34			
**Perceived safety at home**								
Very safe	367	(54.5)	212	(58.4)	155	(41.6)	1.00	
Somewhat safe	244	(38.9)	114	(48.6)	130	(51.4)	1.49	(1.05-2.11)
Not safe at all	47	(6.6)	12	(29.3)	35	(70.7)	3.39	(1.62-7.06)
Missing	3		2		1			
**Number of people to count on in difficult times**								
None	158	(24.2)	90	(57.7)	68	(42.3)	1.00	
One	261	(39.1)	94	(37.7)	167	(62.3)	2.25	(1.46-3.47)
Two to five	227	(34.7)	145	(64.7)	82	(35.3)	0.75	(0.48-1.16)
Six and more	12	(2.0)	10	(87.0)	2	(13.0)	0.20	(0.04-0.99)
Missing	3		1		2			
**Legal residency permit**								
No	520	(79.1)	263	(52.1)	257	(47.9)	1.00	
Yes	132	(20.9)	74	(58.0)	58	(42.0)	0.79	(0.52-1.19)
Missing	9		3		6			

CI, confidence interval; OR, odds ratio; GAD-7, Generalised Anxiety Disorder-7
*GAD-7 score ≥10 indicates having anxiety symptoms.

Table S6. Predictors of depressive and anxiety symptoms among Lebanese adults

PHQ-9 score ≥10*				GAD-7 score ≥10†			
**Model predictors**	**Codes**	**Penalised coefficients**	**aOR (95%CI)**		**Codes**	**Penalised coefficients**	**aOR (95%CI)**
**Education**				**Age**	-	-0.01	0.99 (0.97-1.01)
School not attended	0	0.46	1.00	**Education**			
School not completed	1	-	0.39 (0.18-0.81)	School not attended	0	0.62	1.00
School completed	2	-0.18	0.56 (0.26-1.21)	School not completed	1	-	0.53 (0.30-0.92)
Vocational school	3	-	0.51 (0.27-0.98)	School completed	2	-	0.47 (0.22-0.97)
Higher education	4	-	0.62 (0.36-1.08)	Vocational school	3	-	0.41 (0.19-0.89)
**Number of chronic illnesses**	-	0.05	1.09 (0.93-1.28)	Higher education	4	-0.23	0.36 (0.18-0.72)
**Pain that impacts usual activity**				**Marital status**			
No	0	-	1.00	Single/ Engaged	0	-	1.00
Yes	1	0.68	1.92 (1.31-2.83)	Married	1	-	1.08 (0.72-1.61)
**Eviction notice**				Divorced/ Separated/ Widowed	2	0.15	1.38 (0.75-2.54)
No	0	-0.75	1.00	**Pain that impacts usual activity**			
Yes	1	-	1.79 (0.89-3.60)	No	0	-	1.00
Owned	2	-	4.39 (1.40-13.7)	Yes	1	0.78	2.28 (1.57-3.31)
**Household water insecurity**				**Eviction notice**			
Secure	0	-	1.00	No	0	-0.65	1.00
Insecure	1	0.56	1.92 (1.27-2.90)	Yes	1	-	1.66 (0.82-3.32)
**Household food insecurity**				Owned	2	-	3.21 (1.23-7.94)
Secure	0	-	1.00	**Household water insecurity**			
Insecure	1	1.81	6.18 (3.85-9.93)	Secure	0	-	1.00
**Debt**				Insecure	1	0.18	1.29 (0.88-1.89)
No	0	-	1.00	**Household food insecurity**			
Yes	1	0.91	2.61 (1.83-3.72)	Secure	0	-	1.00
**Perceived safety at home**				Insecure	1	1.69	5.41 (3.51-8.34)
Very safe	0	-0.63	1.00	**Debt**			
Somewhat safe	1	-	1.94 (1.37-2.76)	No	0	-	1.00
Not safe at all	2	0.34	3.62 (1.51-8.66)	Yes	1	1.13	3.14 (2.21-4.46)
**Housing tenure status**				**Perceived safety at home**			
Rented	0	0.89	1.00	Very safe	0	-0.76	1.00
Owned	1	-	0.20 (0.06-0.62)	Somewhat safe	1	-	2.11 (1.50-2.96)
Other/ hosted	2	-0.23	0.15 (0.01-1.26)	Not safe at all	2	0.35	3.53 (1.49-8.34)
**Number of people to count on in difficult times**				**Housing tenure status**			

Zero	0	0.32	1.00	Rented	0	0.51	1.00
One	1	-	0.68 (0.38-1.00)	Owned	1	-	0.38 (0.15-0.96)
Two to five	2	-1.05	0.22 (0.13-0.35)	Other/ hosted	2	-	0.49 (0.12-2.08)
Six or more	3	-	0.83 (0.29-2.40)	**Number of people to count on in difficult times**			
				Zero	0	0.21	1.00
				One	1	-	0.74 (0.46-1.19)
				Two to five	2	-	0.26 (0.16-0.42)
				Six or more	3	-1.03	0.61 (0.21-1.74)
**Intercept**	-2.78			**Intercept**	-1.79		
**Selected penalty strength λ**	0.0142364			**Selected penalty strength λ**	0.0135221		
**AUC**	0.81 (95%CI: 0.78-0.84)			**AUC**	0.80 (95%CI: 0.77-0.83)		
**C-Slope**	1.06 (95%CI: 0.90-1.21)			**C-Slope**	1.02 (95%CI: 0.87-1.17)		
**CITL**	-0.002			**CITL**	0.004		
**E:O**	1.001			**E:O**	0.998		
**Number of complete observations in this model**	1,025			**Number of complete observations in this model**	1,031		

aOR, adjusted odds ratio; AUC, area under the receiver operating characteristics curve; CITL, calibration-in-the-large; C-Slope, calibration-slope; CI, confidence interval; E:O, Expected-to-Observed ratio; PHQ-9, Patient Health Questionnaire-9; GAD-7, Generalised Anxiety Disorder-7

*PHQ-9 score ≥10 indicates having depressive symptoms.

†GAD-7 score ≥10 indicates having anxiety symptoms.

Table S7. Predictors of depressive and anxiety symptoms among Syrian refugees or migrants

PHQ-9 score ≥10*				GAD-7 score ≥10†			
**Model predictors**	**Codes**	**Penalised coefficients**	**aOR (95%CI)**		**Codes**	**Penalised coefficients**	**aOR (95%CI)**
**Education**				**Education**			
School not attended	0	0.81	1.00	School not attended	0	-	1.00
School not completed	1	-	0.44 (0.23-0.84)	School not completed	1	-	0.73 (0.44-1.23)
School completed	2	-0.36	0.25 (0.09-0.72)	School completed	2	-0.32	0.42 (0.18-0.96)
Vocational school	3	-	0.57 (0.14-1.35)	Vocational school	3	-	1.19 (0.37-3.84)
Higher education	4	-	0.69 (0.16-2.90)	Higher education	4	-	0.42 (0.12-1.47)
**Marital status**				**Pain that impacts usual activity**			
Single/ Engaged	0	-	1.00	No	0	-	1.00
Married	1	-	1.10 (0.57-2.09)	Yes	1	0.39	1.55 (1.05-2.27)
Divorced/ Separated/ Widowed	2	0.48	2.06 (0.62-6.79)	**Debt**			
**Number of chronic illnesses**		0.21	1.31 (0.93-1.82)	No	0	-	1.00
**Pain that impacts usual activity**				Yes	1	1.26	2.87 (1.75-4.70)
No	0	-	1.00	**Perceived safety at home**			
Yes	1	0.52	1.69 (1.05-2.70)	Very safe	0	-0.35	1.00
**Eviction notice**				Somewhat safe	1	-	1.29 (0.87-1.91)
No	0	-	1.00	Not safe at all	2	0.91	3.65 (1.67-7.94)
Yes	1	0.45	1.61 (0.97-2.68)	**Number of people to count on in difficult times**			
**Household food insecurity**				Zero	0	-	1.00
Secure	0	-	1.00	One	1	0.50	2.70 (1.66-4.37)
Insecure	1	1.61	5.19 (1.82-14.80)	Two to five	2	-0.65	0.73 (0.44-1.23)
**Debt**				Six or more	3	-	0.40 (0.07-2.13)
No	0	-	1.00				
Yes	1	1.47	4.09 (2.12-7.87)				
**Perceived safety at home**							
Very safe	0	-0.82	1.00				
Somewhat safe	1	-	2.21 (1.36-3.59)				

Not safe at all	2	0.35	3.58 (1.47-8.72)				
**Number of people to count on in difficult times**							
Zero	0	-	1.00				
One	1	0.95	2.67 (1.46-4.90)				
Two to five	2	-1.05	0.32 (0.16-0.60)				
Six or more	3	-	0.96 (0.17-5.28)				
**Legal residency permit**							
No	0	0.23	1.00				
Yes	1	-0.54	0.56 (0.31-1.01)				
**Intercept**	-2.85			**Intercept**	-1.05		
**Selected penalty strength λ**	0.0039403			**Selected penalty strength λ**	0.047804		
**AUC**	0.83 (95%CI: 0.80-0.88)			**AUC**	0.72 (95%CI: 0.68-0.76)		
**C-Slope**	1.02 (95%CI: 0.84-1.19)			**C-Slope**	1.01 (95%CI: 0.79-1.23)		
**CITL**	-0.073			**CITL**	-0.031		
**E:O**	1.028			**E:O**	1.014		
**Number of complete observations in this model**	520			**Number of complete observations in this model**	524		

aOR, adjusted odds ratio; AUC, area under the receiver operating characteristics curve; CITL, calibration-in-the-large; C-Slope, calibration-slope; CI, confidence interval; E:O, Expected-to-Observed ratio; PHQ-9, Patient Health Questionnaire-9; GAD-7, Generalised Anxiety Disorder-7

*PHQ-9 score ≥10 indicates having depressive symptoms.

†GAD-7 score ≥10 indicates having anxiety symptoms.

Figure S2. Calibration plot of anxiety symptoms prediction model among Lebanese adults

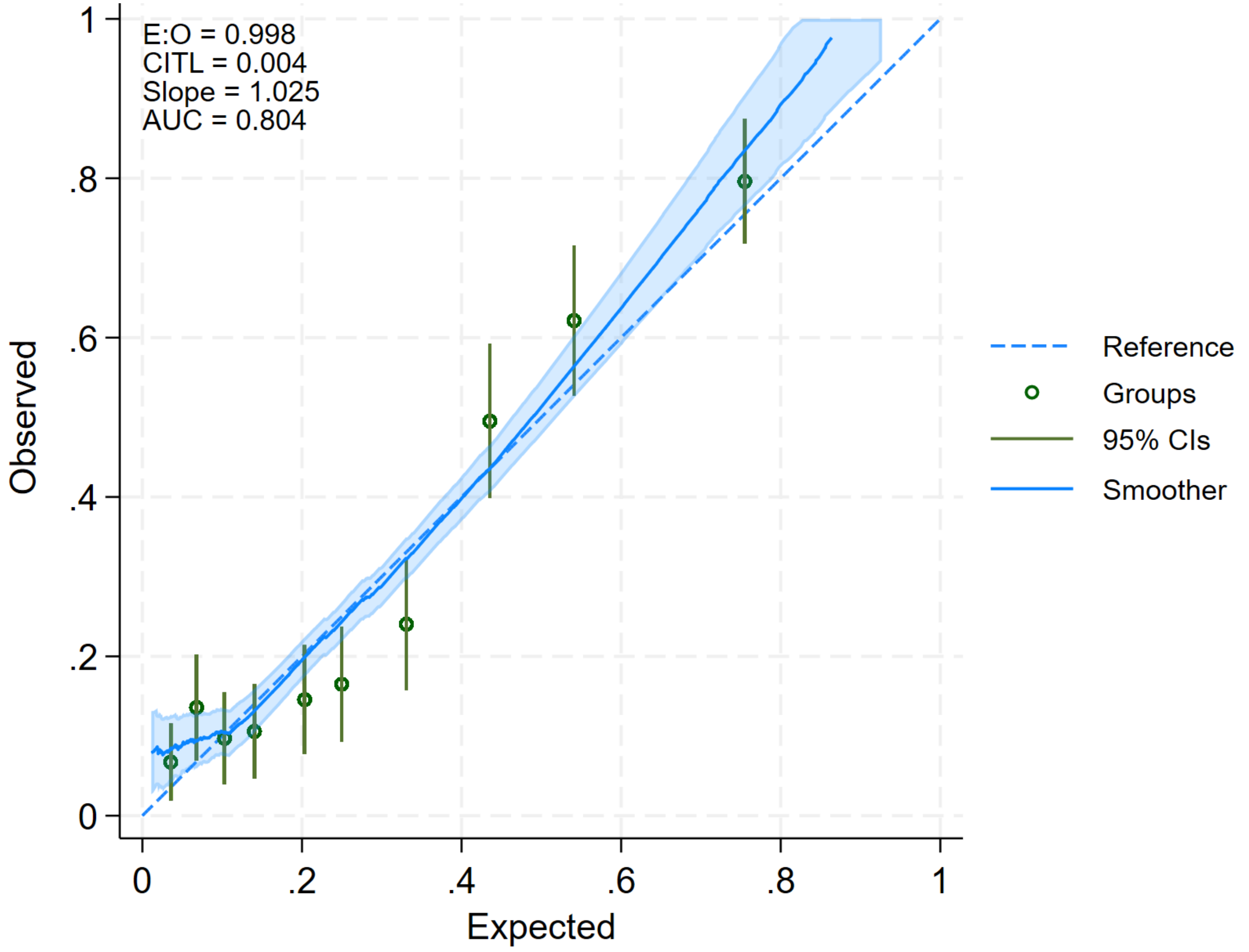

Figure S3. Calibration plot of anxiety symptoms prediction model among Syrian refugees or migrants

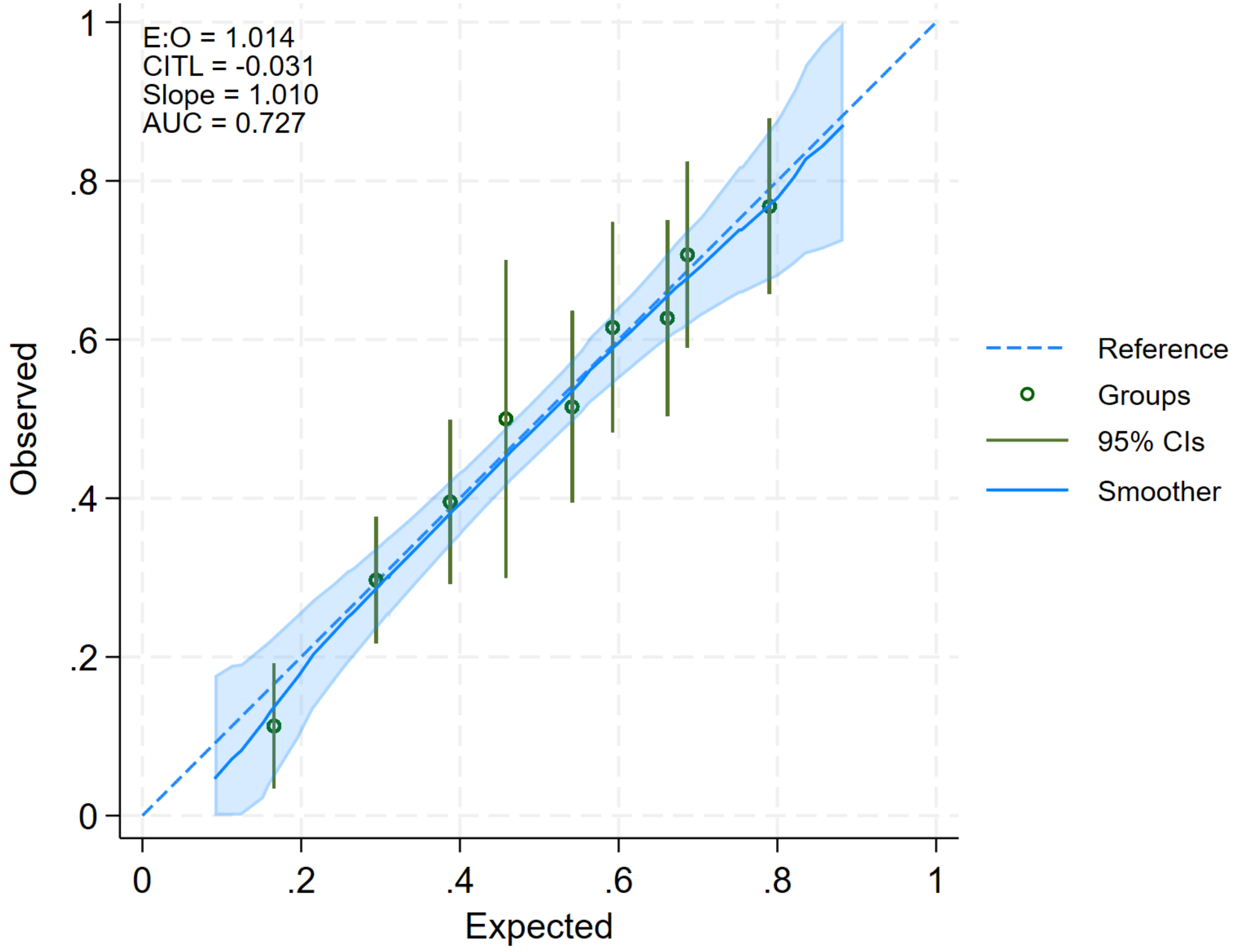

Supplement: online supplemental table 1 [file bmjopen-16-5-s001.pdf]
