# Supplementary material for: Predicting depressive and anxiety symptoms among Lebanese and Syrian adults in a suburb of Beirut, Lebanon, during concurrent crises: nested cross-sectional study
Source: BMJ Open. 2026 May 11;16(5):e101258. doi: 10.1136/bmjopen-2025-101258 (PMC13182334; doi:10.1136/bmjopen-2025-101258)
Supplement: Supplementary file 2 [file bmjopen-2025-101258.reviewer_comments.pdf]

PEER REVIEW HISTORY

BMJ Open publishes all reviews undertaken for accepted manuscripts. Reviewers are asked to complete a checklist review form and are provided with free text boxes to elaborate on their assessment. These free text comments are reproduced below.

ARTICLE DETAILS

Title (Provisional)

Predicting Depressive and Anxiety Symptoms Among Lebanese and Syrian Adults in a Suburb of Beirut, Lebanon During Concurrent Crises: Nested Cross-sectional Study

Authors

Shamas, Hazar; Ragi, Marie-Elizabeth; Abi Zeid, Berthe; DeJong, J; Study Group, CAEP; McCall, Stephen J

VERSION 1 - REVIEW

**Reviewer**	**1**
**Name**	**Javanbakht, Arash**
**Affiliation**	**Wayne State University**
**Date**	**18-May-2025**
**COI**	**None**

This study examines the prevalence of depression and anxiety symptoms among Lebanese citizens and Syrian refugees in Lebanon toward the end of the COVID-19 pandemic and their socioeconomic correlates for each of these populations. During the pandemic time, this work would have novelty to global audience given the immediate impact was being assessed globally. But now, it is unclear what this cross sectional work would add to what we know. Most importantly, this data is noted to be from a longitudinal study. That is a treasure source, which is not used for this work. If the investigators were looking at correlates of who is impacted more by the pandemic, and its SES correlates, using their previous data on this population, the work would be of much higher impact. I am sure this work in its current cross sectional form can find a home, but I believe does not have enough novelty for the BMJ.

Reviewer **2**

Name **Fornaro, Michele**

Affiliation **Section of Psychiatry, University School of Medicine Federico II, Naples, Italy**

**Date**	**02-Jan-2026**
**COI**	**None**

This submission is highly relevant, has a clear public-mental-health rationale focusing on low-SES host communities and refugees during overlapping crises, and is timely and essential. I appreciated the pragmatic candidate predictors (financial strain, food/water insecurity, safety, pain, chronic illness, social support) are actionable at a policy/service level, transparent intent to follow reporting guidance (TRIPOD/STROBE noted), and depression model performance is promising as reported for PHQ-9≥10 in both Lebanese and Syrian samples (AUC ~0.81 and ~0.83; calibration slopes ~1.06 and ~1.02). There are a few issues to address, nonetheless:

1) Your outcomes (PHQ-9 ≥ 10; GAD-7 ≥ 10) and predictors are collected at the same telephone survey time point. That makes these concurrent classification models, not risk/prognostic models. The manuscript should either: reframe as concurrent correlates/classification/risk scoring for current symptoms, or clearly justify how the tool would be used before symptom assessment (otherwise, the PHQ-9/GAD-7 are already brief screens). If the goal is case-finding, explain what the model adds beyond simply administering the PHQ-9/GAD-7 (9 and 7 items), especially since the model already uses multiple socioeconomic questions.

2) You state missingness was "missing at random," test missingness using indicators/logit models, but then use complete-case analysis. For MAR, complete cases can bias estimates and degrade transportability. Please report % missing per predictor, number of full cases per model, and strongly consider multiple imputation (or at a minimum, sensitivity analyses comparing complete-case vs imputed). If missing indicators were created, clarify whether they were included/excluded from the LASSO step.

3) You report "adaptive LASSO logistic regression" with all candidate predictors entered, and then run separate logistic regressions on selected predictors for ORs. I think that there are some key omissions anyway: How was λ (penalty strength) chosen (CV? 1-SE rule?). How was adaptive LASSO implemented (initial weights from which model? how computed)? How were survey weights/complex sampling incorporated into penalized estimation? Your state analyses were weighted for complex design/non-response, but penalized survey-weighted logistic regression is non-trivial and needs explicit methods/software details. Post-selection "refit" ORs: refitting unpenalized models on selected predictors can inflate performance and produce optimistic/unstable inference (selection-induced bias). If you want interpretability, consider reporting penalized coefficients + intercept as the primary "model," and use refit ORs cautiously (or use post-selection inference approaches).

4) The abstract claims "develop and internally validate." In the methods/results, performance is reported (AUC, calibration slope, plots), but the validation procedure (bootstrap optimism correction, cross-validation, split-sample, etc.) is not clearly described.

Please consider adding a dedicated paragraph: Internal validation method (prefer bootstrap resampling for optimism-corrected AUC/calibration in prediction models). Report apparent vs optimism-corrected performance.

5) You emphasize AUC and C-slope. For implementability, also report: Calibration intercept, Brier score, and/or E/O ratio. Clinically relevant threshold metrics (sensitivity/specificity/PPV/NPV at one or two plausible cut points). Ideally decision curve analysis (net benefit), because these tools will likely guide referral/resource allocation. Also: anxiety model metrics are relegated to supplemental text/figures ("model evaluation metrics are presented in the results section of the supplemental…"). At least provide the AUC and calibration slope for the anxiety models in the main manuscript to ensure symmetry and readability.

6) You dichotomize PHQ-9 and GAD-7 at ≥10. Given two groups (Lebanese vs. Syrian refugees) and likely language/cultural differences, you should discuss: versions used (Arabic? validated locally?); mode (telephone); and potential differential item functioning/measurement invariance, whether cutoffs have similar operating characteristics in both groups.

7) Sampling is from low-SES neighborhoods in Sin-El-Fil with telephone survey recruitment. You should be explicit that the model is likely context-bound and requires external validation before broader application. Also, using legal residency documentation as a predictor (and recommending advocacy around it) is essential. Still, if the model were used operationally, legal status variables can create risks of stigma or unintended harms. The manuscript should add an ethics paragraph on: Safeguards against using the score for exclusion/penalization. Ensuring the model supports supportive outreach, not surveillance.

8) Table formatting/typos: In Supplemental Table S6, "Having debt… OR 261 (1.83–3.72)" appears to be a missing decimal (likely 2.61). Clarify variable coding where tables show categories like "Owned" under eviction notice/tenure—some labels are confusing. Please provide the complete final model equation (including the intercept) for each of the four models, not just a list of retained predictors. The supplemental tables contain penalized coefficients, but implementers still need a complete scoring rule. Finally, please consider citing this in the introduction: PMID: 39706484 for background/rationale: to support the general claim that armed conflict and mass displacement are associated with increased mental health burden and unmet service needs, especially among vulnerable groups (including refugees), and that evidence gaps remain. And in the discussion contextualization: to frame Lebanese/Syrian findings within the broader conflict-migration mental health literature and to justify the need for scalable screening/triage approaches in humanitarian settings.

VERSION 1 - AUTHOR RESPONSE

Reviewer: 1
Dr. Arash Javanbakht, Wayne State University
Comments to the Author:
This study examines the prevalence of depression and anxiety symptoms among Lebanese citizens and Syrian refugees in Lebanon toward the end of the COVID-19 pandemic and their socioeconomic correlates for each of these populations. During the pandemic time, this work would have novelty to global audience given the immediate impact was being assessed globally. But now, it is unclear what this cross sectional work would add to what we know. Most importantly, this data is noted to be from a longitudinal study. That is a treasure source, which is not used for this work. If the investigators were looking at correlates of who is impacted more by the pandemic, and its SES correlates, using their previous data on this population, the work would be of much higher impact. I am sure this work in its current cross sectional form can find a home, but I believe does not have enough novelty for the BMJ.

Response: We thank the reviewer very much for these comments. This study was a cross-sectional analysis to identify the stressors of mental health disorders among Syrian refugees and migrants and host population during overlapping crises in Lebanon. Within the duration of data collection from June – October 2022 in Sin-El-Fil, Beirut, Lebanon, the country was simultaneously experiencing concurrent crises including the impact of the Beirut port blast, prolonged political instability, COVID-19 pandemic, and economic crisis. Moreover, to our knowledge, studies examining predictors of mental health disorders in Lebanon were restricted to either older Syrian refugees only, widowed Syrian refugees only, or Syrian refugees who experienced ambiguous loss of a close family member (1-3). The data in this study were robustly analysed and reported where this is the first study to examine predictors of having depressive and anxiety symptoms among Syrian refugees and host community during concurrent crises.
We thank the reviewer for the valuable idea, the research team will consider it for future study.

References:
1- Abi Zeid B, Farouki L, El Khoury T, Sibai A, Mendes de Leon CF, Alawieh M, et al. Predicting poor mental health amongst older Syrian refugees in Lebanon during the COVID-19 pandemic: a multi-wave longitudinal study. medRxiv. 2023:2023.12. 22.23300447.
2- Hosseini Z, Bakdash T, Ahmad S, Awaad R. Predictors of depression among Syrian refugee women: A socio-culturally relevant analysis. International Journal of Social Psychiatry. 2023:00207640231155810.
3- Renner A, Jäckle D, Nagl M, Plexnies A, Röhr S, Löbner M, et al. Traumatized Syrian refugees with ambiguous loss: Predictors of mental distress. International Journal of Environmental Research and Public Health. 2021;18(8):3865.

***editor's note: novelty is not an essential criteria for being considered for publication with BMJ Open. Please also note that any suggestions to change the study design at this stage should be rebutted rather than adhered to.

Reviewer: 2
Dr. Michele Fornaro, Section of Psychiatry, University School of Medicine Federico II, Naples,

Italy, Columbia University
Comments to the Author:
This submission is highly relevant, has a clear public-mental-health rationale focusing on low-SES host communities and refugees during overlapping crises, and is timely and essential. I appreciated the pragmatic candidate predictors (financial strain, food/water insecurity, safety, pain, chronic illness, social support) are actionable at a policy/service level, transparent intent to follow reporting guidance (TRIPOD/STROBE noted), and depression model performance is promising as reported for PHQ-9≥10 in both Lebanese and Syrian samples (AUC ~0.81 and ~0.83; calibration slopes ~1.06 and ~1.02). There are a few issues to address, nonetheless:

Response: We thank the reviewer very much for these comments. They have been addressed in the points below.

1) Your outcomes (PHQ-9 ≥ 10; GAD-7 ≥ 10) and predictors are collected at the same telephone survey time point. That makes these concurrent classification models, not risk/prognostic models. The manuscript should either: reframe as concurrent correlates/classification/risk scoring for current symptoms, or clearly justify how the tool would be used before symptom assessment (otherwise, the PHQ-9/GAD-7 are already brief screens). If the goal is case-finding, explain what the model adds beyond simply administering the PHQ-9/GAD-7 (9 and 7 items), especially since the model already uses multiple socioeconomic questions.

Response: We thank the reviewer for this comment. PHQ-9 is the gold standard for screening. The aim of this study was to explore multiple socioeconomic (SES) factors that predict low/high PHQ-9. Thus, our aim was to identify the risk profiles of vulnerability to low/high PHQ-9. This is valuable for public health planning in a setting with limited resources where universal screening may not be feasible. As such, it provides a clear public health message of targeting and improving social interventions may mitigate the burden of mental health disorders.
Given the scarcity of population-based data in Lebanon and the absence of an external dataset to validate our prediction models, we did not externally validate the developed models. Thus, we refrained from calculating individual risk scores of having depressive or anxiety symptoms among our study population.
Moreover, in accordance with prior studies in the literature, we framed this analysis as a prediction (classification) modelling approach aimed at identifying individuals with current symptoms rather than predicting future risk.

References:
1- Smith J, Lee A, Zhao Q, et al. Machine learning-based predictive modeling of depressive symptoms using lifestyle indicators and socioeconomic status: a cross-sectional study. *Journal of Affective Disorders Reports*. 2025;5(2):123–130.
2- Kożybska M, Wojtkowska A, Szpakow A, Fedortsiv O, et al. Predicting depressive symptoms among Polish university students through sociodemographic and lifestyle factors. *Scientific Reports*. 2025;15:43959. doi:10.1038/s41598-025-27728-w
3- Dai Z, Liu X, Jing S, Wang H, Huang Y, Fu J, Wu Y, Zhang L, Han B, Su X. Development and internal validation of a depressive symptoms prediction model among patients with

cardiovascular disease who have recovered from SARS-CoV-2 infection in Wuhan, China: a cross-sectional study. *BMC Psychiatry*. 2025;25(1):492. doi:10.1186/s12888-025-06886-1.

2) You state missingness was "missing at random," test missingness using indicators/logit models, but then use complete-case analysis. For MAR, complete cases can bias estimates and degrade transportability. Please report % missing per predictor, number of full cases per model, and strongly consider multiple imputation (or at a minimum, sensitivity analyses comparing complete-case vs imputed). If missing indicators were created, clarify whether they were included/excluded from the LASSO step.

Response: We thank the reviewer for this comment. The number of complete cases per model were added into Tables S6 and S7 pages 11-14 of the Supplemental Appendix. The number of missing values per candidate predictor were reported in Tables 1 and 2 of the manuscript pages 16-19 and Table S4 and S5 pages 7-10 of the Supplemental Appendix. Please note that unweighted counts of missing data rather than percentages were reported, as descriptive tables report weighted percentages following standard practice.
We explored multiple imputation extensively during the analysis. However, several continuous and categorical predictors contained sparse or empty categories that resulted in quasi-complete or complete separation in the imputation models. Addressing this issue would require collapsing continuous variables into categories or altering variable definitions which would change the interpretation of key predictors.
Given the very small number of missing data (maximum of 8% in one variable) and given that forcing an imputation model on sparse data risks introducing its own bias, we prioritised the original measurement structure. Thus, we retained a complete-case analysis as the primary approach for this paper, with extensively reporting on missingness.

Indicators developed for testing missingness were created only to explore patterns of missing data and assess the plausibility of MAR assumptions. Thus, these indicators were not included in the adaptive LASSO variable selection step. The methods section was revised as follows:

Methods *Candidate predictors* section page 7, lines 185-190:
"To explore the pattern of missingness only among candidate predictors, an indicator variable for each existing candidate predictor with missing data was generated. A value of 1 was given for every missing observation and a value of 0 was given if an observation was not missing. Logit models were then run to test if any of the candidate predictors could predict whether a given observation was missing (25). Results indicated that the missing values were missing at random, and a complete-case analysis was implemented."

3) You report "adaptive LASSO logistic regression" with all candidate predictors entered, and then run separate logistic regressions on selected predictors for ORs. I think that there are some key omissions anyway:
How was λ (penalty strength) chosen (CV? 1-SE rule?).
How was adaptive LASSO implemented (initial weights from which model? how computed)?
How were survey weights/complex sampling incorporated into penalized estimation?
Your state analyses were weighted for complex design/non-response, but penalized survey-weighted logistic regression is non-trivial and needs explicit methods/software details.

Post-selection "refit" ORs: refitting unpenalized models on selected predictors can inflate performance and produce optimistic/unstable inference (selection-induced bias). If you want interpretability, consider reporting penalized coefficients + intercept as the primary "model," and use refit ORs cautiously (or use post-selection inference approaches).

Response: We thank the reviewer for this comment.
The penalty strength λ was selected using 10-fold cross-validation. The dataset was partitioned into 10 folds, with models iteratively trained on nine folds and evaluated on the remaining fold. The λ value that minimised the mean cross-validated prediction error was retained for the final model. The following was added:

Methods *Statistical analysis* section page 8, lines 210-213:
"The penalty strength (λ) was selected using 10-fold cross-validation, where the dataset was partitioned into 10 folds, with models iteratively trained on nine folds and evaluated on the remaining fold. The λ value that minimised the mean cross-validated prediction error was retained for the final model."

Adaptive LASSO logistic regression was used for model development. In this approach, an initial standard penalised regression model is fitted to obtain preliminary coefficient estimates, which are then used to construct variable-specific penalty weights. A penalty-weighted LASSO model is subsequently refitted, allowing differential shrinkage across predictors. The optimal penalty parameter (λ) was selected using 10-fold cross-validation to minimise cross-validated deviance. The following was added:
Methods *Statistical analysis* section page 7, lines 205-210:
"Adaptive Least Absolute Shrinkage and Selection Operator (LASSO) logistic regression model was implemented to identify the predictors of depressive and anxiety symptoms for Lebanese and Syrians. For this approach, an initial standard penalised regression model is fitted to obtain preliminary coefficient estimates, which are then used to construct variable-specific penalty weights. A penalty-weighted LASSO model is subsequently refitted, allowing differential shrinkage across predictors."

The study weights were computed as the product of the baseline sampling weight (inverse probability of selection in the study sample) and the non-response adjustment factor. The non-response weight was estimated using a response propensity logistic regression model in which response status was modelled as a function of observed covariates to account for attrition during data collection. However, given the complexities of incorporating survey weights into penalised estimation due to software limitation and the potential for increased variance in the selected model, we utilised study weights for the descriptive estimates but utilised penalty-weighted data only for the variable selection and model development steps. For further details on the computation of the study weights, please refer to the Supplemental Appendix of our following study publication:

Reference:
1- Fayyad R, Ragi M, Ghattas H, McCall SJ. Social determinants of self-reported health in vulnerable populations during a polycrisis in Lebanon. JAMA Netw Open. 2025;8(10):e2529733.

The following was revised in the manuscript:

Methods *Statistical analysis* section, page 7 lines 194-200:
"The study weights were calculated as the product of the baseline sampling weight (inverse probability of selection in the study sample) and the non-response adjustment factor. The non-response weight was estimated using a response propensity logistic regression model in which response status was modelled as a function of observed covariates to account for attrition during data collection. Further description of study weight computation is presented elsewhere (20). Study weights were used for descriptive estimates only and penalty weights were used for variable selection and model development."

We have clarified in the text that penalised models represent the primary models while the adjusted ORs from the refitted logistic regression models are provided solely to aid in the clinical interpretation of the magnitude of associations for the selected subset of predictors. All penalised coefficients and intercepts were reported in Tables S6 and S7 in the Supplemental Appendix pages 11-14.
The following was added:

Methods *Statistical analysis* section page 8, lines 213-214:
"The penalised models represent the primary analysis, and the coefficients and intercept of the final developed models were reported."

4) The abstract claims "develop and internally validate." In the methods/results, performance is reported (AUC, calibration slope, plots), but the validation procedure (bootstrap optimism correction, cross-validation, split-sample, etc.) is not clearly described. Please consider adding a dedicated paragraph: Internal validation method (prefer bootstrap resampling for optimism-corrected AUC/calibration in prediction models). Report apparent vs optimism-corrected performance.

Response: We thank the reviewer for this comment. The model was internally validated using 10-fold cross-validation for parameter tuning of λ, where the data set was randomly divided into 10 folds. In each iteration, 9 folds were used for training the model while the remaining fold served as the validation set. Model performance was averaged across the 10 validation folds to obtain cross-validated estimates of discrimination (AUC) and calibration.

Based on the reviewer's comment, bootstrap resampling with 1,000 samples was conducted on the developed prediction models and there was no difference in the model performance. Thus, we have kept the modelled parameters form the original model.

The objective in the abstract was edited as follows:

Abstract section page 2, lines 34-36:
"This study aimed to develop prediction models for having depressive and anxiety symptoms among Lebanese adults and Syrian refugees or migrants residing in a suburb of Beirut, Lebanon."

5) You emphasize AUC and C-slope. For implementability, also report: Calibration intercept, Brier score, and/or E/O ratio.
Clinically relevant threshold metrics (sensitivity/specificity/PPV/NPV at one or two plausible cut points).
Ideally decision curve analysis (net benefit), because these tools will likely guide referral/resource allocation.
Also: anxiety model metrics are relegated to supplemental text/figures ("model evaluation metrics are presented in the results section of the supplemental…"). At least provide the AUC and calibration slope for the anxiety models in the main manuscript to ensure symmetry and readability.

Response: We thank the reviewer for the constructive comment regarding model performance reporting. The calibration-in-the-large (CITL) and the Expected-to-Observed ratio (E:O) were reported in Tables S6 and S7 of the Supplemental Appendix pages 11-14 as well as in the calibration plots Figures 1 and 2 of the manuscript, and Figures S1 and S2 in the Supplemental Appendix pages 15 and 16. The following was also added in the manuscript:
Methods *Statistical analysis* section page 8, lines 220-223.
"The calibration-in-the-large (CITL) was computed to compare the observed number of events and the average predictive risk. The Expected-to-Observed ratio (E:O), was calculated as the ratio of the expected number of events (sum of predicted probabilities) to the observed number of events."

As noted earlier, the primary aim of this study was to identify common and disparate stressors of depressive and anxiety symptoms to inform service delivery and public health planning rather than clinical prediction for individual risk stratification or immediate triage, thus the decision curve analysis was not performed. As such, we focused on the global measures of model performance rather than defining probability thresholds of net benefit, which would require defining theoretical intervention consequences that are beyond the scope of this study. We kindly defer to the editor on this point. The sensitivity and 1−specificity across all possible probability thresholds are visualised in the ROC curves Figures 1 and 2 of the manuscript and Figures S1 and S2 in the Supplemental Appendix pages 15 and 16.

We have moved the AUC and calibration slopes for the anxiety models into main text. The following was added:

Results page 9, lines 240-258:
"The Lebanese model predicting depressive symptoms retained ten predictors: education, number of chronic illnesses, pain that impacted usual activity, eviction notice, household water insecurity, household food insecurity, having debt, feeling safe inside home, type of housing tenure, and number of people to count on in difficult times. This model had good discrimination (AUC:0.81; 95%CI: 0.78-0.84), excellent calibration (C-Slope:1.06; 95% CI: 0.90-1.21), CITL of -0.002, and E:O of 1.001 (Figure 1, Supplemental Table 6).
Predictors of the Lebanese model predicting anxiety symptoms are presented in Supplemental Table 6. Similarly, the model had good discrimination (AUC:0.80; 95%CI: 0.77-

0.83), excellent calibration (C-Slope:1.02; 95%CI: 0.87-1.17), CITL of 0.004, and E:O of 0.998 (Supplemental Figure 1 and Supplemental Table 6).

The Syrian model predicting depressive symptoms retained 10 predictors: education, marital status, number of chronic diseases, pain that impacted usual activity, eviction notice, household food insecurity, having debt, feeling safe inside home, number of people to count on in difficult times, and legal status documentation. This model had good discrimination (AUC:0.83; 95%CI: 0.80-0.88), good calibration slope (C-Slope:1.02; 95%CI:0.84-1.19), CITL of -0.073, and E:O of 1.028 (Figure 2, Supplemental Table 7).

Predictors of the Syrian model predicting anxiety symptoms is presented in Supplemental Table 7. Similarly, the model had good discrimination (AUC:0.72; 95%CI: 0.68-0.76), excellent calibration (C-Slope:1.01; 95%CI: 0.79-1.23), CITL, -0.031, and E:O of 1.014 (Supplemental Figure 2 and Supplemental Table 7)."

References:

1- Çorbacıoğlu SK, Aksel G. Receiver operating characteristic curve analysis in diagnostic accuracy studies: a guide to interpreting the area under the curve value. Turk J Emerg Med. 2023;23(4):195–198.
2- Vickers AJ, Elkin EB. Decision curve analysis: a novel method for evaluating prediction models. *Med Decis Making.* 2006;26(6):565–574.
3- Steyerberg EW. Clinical Prediction Models: A Practical Approach to Development, Validation, and Updating. 2nd ed. Cham: Springer; 2019.

6) You dichotomize PHQ-9 and GAD-7 at ≥10. Given two groups (Lebanese vs. Syrian refugees) and likely language/cultural differences, you should discuss: versions used (Arabic? validated locally?); mode (telephone); and potential differential item functioning/measurement invariance, whether cutoffs have similar operating characteristics in both groups.

Response: We thank the reviewer for this comment. The scales PHQ-9 and GAD-7 were validated in Arabic locally. We believe that the construct is similar because of shared culture, context, and language so a differential item functioning analysis was not deemed as necessary by the study team. Furthermore, the internal reliability (Cronbach's alpha) was excellent for the total sample (0.89 for PHQ-9 and 0.95 for GAD-7), suggesting the constructs were measured consistently. The following was added into the limitations:

<u>Discussion page 12 lines 324-327:</u>
"The measurement invariance was not formally tested across nationality. However, both groups share Arabic as a common language, and substantial cultural and social overlap within the same geographic context, which may reduce the likelihood of major construct-level differences."

The PHQ-9 and GAD-7 are robust to different modes of administration including telephones. While Arabic validation may have been conducted by face to face interviews, collecting data through the telephone is common practice in Lebanon. Additionally, during the pandemic and crises, it became necessary to conduct interviews via telephone. Thus, we trained data

collectors a priori to ensure standardised administration of scales to minimise data collection mode-induced biases. Moreover, we added the following into the limitation section:

Discussion page 11, lines 322-324:
"While PHQ-9 and GAD-7 have been rigorously validated in Arabic for face-to-face and self-report formats, specific psychometric validation of their Arabic versions when administered via telephone remains limited."

References:
1- Pinto-Meza A, Serrano-Blanco A, Peñarrubia MT, Blanco E, Haro JM. Assessing depression in primary care with the PHQ-9: Can it be carried out over the telephone? J Gen Intern Med. 2005;20(8):738-42. doi:10.1111/j.1525-1497.2005.0144.x
2- Al-Habeeb AA, Al-Duraihem RA, Alasmary S, Alkhamaali Z, Althumiri NA, BinDhim NF. National screening for anxiety and depression in Saudi Arabia 2022. Front Public Health. 2023;11:1213851. doi:10.3389/fpubh.2023.1213851

7) Sampling is from low-SES neighborhoods in Sin-El-Fil with telephone survey recruitment. You should be explicit that the model is likely context-bound and requires external validation before broader application. Also, using legal residency documentation as a predictor (and recommending advocacy around it) is essential.
Still, if the model were used operationally, legal status variables can create risks of stigma or unintended harms. The manuscript should add an ethics paragraph on: Safeguards against using the score for exclusion/penalization. Ensuring the model supports supportive outreach, not surveillance.

Response: We thank the reviewer for this comment. The aim of developing the prediction models was to identify the common and disparate stressors of mental disorders between Lebanese and Syrians to inform service delivery and public health planning rather than being used operationally. The following was edited in the limitations page 11 of the manuscript:

Discussion pages 11 and 12, lines 318-322:
"The model was developed using data from low SES neighbourhoods in Sin-El-Fil, Lebanon, recruited through a telephone survey. Thus, these findings and model performance are context-specific and reflect the local social, economic, and service environment. Future research should validate these models on datasets in other populations and contexts."

The following was added into the manuscript to safeguard against using models including legal residency as predictors for exclusion/ penalisation:

Discussion page 11, lines 308-314:
"The inclusion of structural variables such as legal residency documentation captures broader determinants of vulnerability. However, the operational use of such models requires careful ethical consideration. Such models should strictly support supportive outreach, resource allocation, and service prioritisation. Clear governance frameworks, transparency in model application, and safeguards against discriminatory use are essential to minimise stigma and unintended harms."

8) Table formatting/typos: In Supplemental Table S6, “Having debt… OR 261 (1.83–3.72)” appears to be a missing decimal (likely 2.61).

Clarify variable coding where tables show categories like “Owned” under eviction notice/tenure—some labels are confusing.

Please provide the complete final model equation (including the intercept) for each of the four models, not just a list of retained predictors. The supplemental tables contain penalized coefficients, but implementers still need a complete scoring rule.

Finally, please consider citing this in the introduction: PMID: 39706484 for background/rationale: to support the general claim that armed conflict and mass displacement are associated with increased mental health burden and unmet service needs, especially among vulnerable groups (including refugees), and that evidence gaps remain.

And in the discussion contextualization: to frame Lebanese/Syrian findings within the broader conflict-migration mental health literature and to justify the need for scalable screening/triage approaches in humanitarian settings.

Response: We thank the reviewer for this comment. The manuscript has been carefully proofread, and the identified typo errors have been corrected throughout.
All model intercepts were added into Tables S6 and S7 pages 11-14 of the Supplemental Appendix.
The variable coding “Owned” was relabelled as “Housing tenure status” in Manuscript Table 1 page 18, and in Tables S4 and S6 of the Supplemental Appendix page 8 and 11.
The reference PMID: 39706484 is highly relevant to the present study. Thus, it was cited in the introduction and discussion sections of the paper. The following was also added into the discussion.

Discussion page 12, lines 331-336:
“Within the broader conflict-migration mental health literature, there is increasing recognition that resource-constrained humanitarian settings require pragmatic, scalable approaches to identify individuals at highest risk (10, 13, 14). Developing and evaluating prediction models for depressive and anxiety symptoms using the PHQ-9 and GAD-7 may support targeted screening and triage strategies, enabling more efficient allocation of limited mental health resources while reflecting broader determinants of vulnerability in these populations (10, 13, 14).”

VERSION 2 - REVIEW

Reviewer 2

Name Fornaro, Michele

Affiliation Section of Psychiatry, University School of Medicine Federico II, Naples, Italy

Date **08-Mar-2026**

COI

My main suggestion is that you should be more precise and restrained in how you describe the models and their implications. The outcomes are depressive symptoms and anxiety symptoms defined by PHQ-9 and GAD-7 cutoffs, not clinical diagnoses of depressive or anxiety disorders. This distinction matters throughout the title, abstract, discussion, and conclusion. In several places, the language moves from symptoms to mental health disorders more generally. I would recommend revising this wording consistently so that the claims remain fully aligned with the measures actually used.

Second, although the manuscript is framed as a prediction study, the interpretation at times becomes close to a causal explanation. Variables such as debt, food insecurity, pain, insecurity at home, and social support are highly plausible correlates of symptom burden, but the cross-sectional design does not allow temporal ordering to be established. Please make this limitation more explicit, especially when discussing targeted intervention and service planning. The models may help identify individuals at a higher current probability of screening positive, but they do not establish that the retained predictors are causes of later symptoms.

Third, the reporting of model development is generally clear, but a few points would benefit from clarification. You state that complete-case analysis was used after assessing the pattern of missingness among candidate predictors. Given the visible amount of missing data for some variables in the tables, please report more explicitly how many participants were included in each final model and whether complete-case exclusion materially changed the effective sample size or events-per-parameter ratio. A short clarification here would help readers judge robustness.

Fourth, I encourage you to moderate the use of terms such as "excellent calibration" and "innovative machine learning tools". Adaptive LASSO is an appropriate and useful penalised regression approach, but the paper will read as stronger if the language is slightly less promotional and more methodologically neutral. Similarly, because calibration was assessed internally rather than through external validation, it would be better to avoid overstatement and to emphasise that the models now require validation in independent Lebanese and Syrian samples before broader implementation.

Fifth, please comment more directly on generalisability. The study is well justified as a context-specific analysis of low socioeconomic neighbourhoods in Sin-El-Fil, but the abstract and conclusion should reiterate that these models are most applicable to similar urban, crisis-affected, low-resource settings rather than to all Lebanese or Syrian populations in Lebanon. This point is already acknowledged, but it deserves slightly more prominence because readers may otherwise overextend the findings.

I also have a few minor suggestions for the presentation. Please ensure complete consistency in terminology for Syrians, for example, refugees or migrants, across the manuscript. Please check the wording around legal residency documentation, so the operational definition is immediately clear to an international readership. In addition, some references used in the discussion appear less authoritative than others for a high-impact public health journal, so I encourage you to review the reference list and, where possible, prioritise peer-reviewed or institutional sources.

VERSION 2 - AUTHOR RESPONSE

Reviewer: 2
Dr. Michele Fornaro, Section of Psychiatry, University School of Medicine Federico II, Naples, Italy, Columbia University

Response: We would like to thank the reviewer for these constructive comments on the manuscript which greatly improved and strengthened the paper. Please find below a point-by-point response to the comments written in blue. All edits have been made on the manuscript. You will kindly find both "track changes" and "clean" versions of the revised manuscript and supplementary appendix, as well as the completed revised clean versions of the STROBE and TRIPOD checklists, attached for your kind review.

Comments to the Author:
My main suggestion is that you should be more precise and restrained in how you describe the models and their implications. The outcomes are depressive symptoms and anxiety symptoms defined by PHQ-9 and GAD-7 cutoffs, not clinical diagnoses of depressive or anxiety disorders. This distinction matters throughout the title, abstract, discussion, and conclusion. In several places, the language moves from symptoms to mental health disorders more generally. I would recommend revising this wording consistently so that the claims remain fully aligned with the measures actually used.

Response: We thank the reviewer for this comment. The wording from mental health disorders to depressive and anxiety symptoms has been consistently revised throughout the manuscript and supplemental material. We have also revised and used the term "poor mental health" instead of mental health disorders in the Abstract and Introduction. Examples of the changes are included the following:

Abstract page 2, lines 34-36:

"Objective: This study aimed to develop prediction models for symptoms of poor mental health among Lebanese adults and adult Syrian refugees or migrants residing in a suburb of Beirut, Lebanon, separately."

Abstract page 2, lines 54-58:

"Conclusions: The predictors of poor mental health were financial, health, and social indicators for both Lebanese adults and Syrian refugees or migrants during the concurrent

crisis in Lebanon. These models are most applicable in similar urban, crisis-affected, and low-resource settings. Findings emphasise the need for social protection and financial support among population with vulnerabilities."

Introduction page 4, lines 104-106:

"Host populations with low SES, refugees, and migrants have stressors that place them at greater risk of poor mental health, including lack of employment opportunities, limited social mobility, and lack of healthcare access [9,10]."

Introduction pages 4 and 5, lines 113-119:

"In the context of Lebanon, there remains an opportunity to explore the predictors of poor mental health amongst both host and refugee populations with the aim of identifying the common and disparate predictors of poor mental health to inform service delivery and public health planning. Therefore, the aim of this study is to develop separate prediction models for depressive and anxiety symptoms among Lebanese adults and adult Syrian refugees or migrants residing in low socioeconomic areas, of a suburb in Beirut, Lebanon."

Discussion page 10, lines 274-278:

"These models identified common and disparate stressors of depressive and anxiety symptoms among Lebanese adults and Syrian refugees or migrants residing in Sin-El-Fil. The findings of this study also showed high prevalence of depressive and anxiety symptoms and several predictors of poor mental health were common between both populations residing in low SES areas in Sin-El-Fil."

Second, although the manuscript is framed as a prediction study, the interpretation at times becomes close to a causal explanation. Variables such as debt, food insecurity, pain, insecurity at home, and social support are highly plausible correlates of symptom burden, but the cross-sectional design does not allow temporal ordering to be established. Please make this limitation more explicit, especially when discussing targeted intervention and service planning. The models may help identify individuals at a higher current probability of screening positive, but they do not establish that the retained predictors are causes of later symptoms.

Response: We thank the reviewer for this comment. The following was added into the limitations section:

Discussion page 12, lines 343-345:

"The models help identify individuals at a higher current probability of screening positive for having depressive and anxiety symptoms and support targeted screening and service planning. However, the retained predictors should not be interpreted as causal."

Third, the reporting of model development is generally clear, but a few points would benefit from clarification. You state that complete-case analysis was used after assessing the

pattern of missingness among candidate predictors. Given the visible amount of missing data for some variables in the tables, please report more explicitly how many participants were included in each final model and whether complete-case exclusion materially changed the effective sample size or events-per-parameter ratio. A short clarification here would help readers judge robustness.

Response: We thank the reviewer for this comment. The number of participants included for each final model along with the observed-to-expected (E:O) ratio were reported in the Supplemental Tables 6 and 7. Exclusion of observations with missing data did not materially alter the effective sample size or the events-per-parameter ratio, and the E:O ratio remained unchanged across models. The following edits were made into the manuscript:

Methods *Statistical analysis* section page 8, lines 226-229:

"Missing values were missing at random and a complete-case analysis was implemented. Further information on the impact of missing data and complete-case analysis is presented in the methods section of the Supplemental Appendix."

Supplemental Appendix *Methods section* page 5, lines 111-122:

"Analysis of missingness pattern

To explore the pattern of missingness among candidate predictors only, an indicator variable for each existing candidate predictor with missing data was generated. A value of 1 was given for every missing observation and a value of 0 was given if an observation was not missing. Logit models were then run to test if any of the candidate predictors could predict whether a given observation was missing [1].

Impact of missing data and complete-case analysis

The exclusion of observations with missing data did not alter the effective sample size or the events-per-parameter ratio.

References:

1- University of Wisconsin-Madison. Multiple Imputation in Stata: Deciding to Impute 2013 [online]. 13 Jan 2013. https://sscc.wisc.edu/sscc/pubs/stata_mi_decide.htm (accessed 7 Jul 2024)."

Fourth, I encourage you to moderate the use of terms such as "excellent calibration" and "innovative machine learning tools". Adaptive LASSO is an appropriate and useful penalised regression approach, but the paper will read as stronger if the language is slightly less promotional and more methodologically neutral. Similarly, because calibration was assessed internally rather than through external validation, it would be better to avoid overstatement and to emphasise that the models now require validation in independent Lebanese and Syrian samples before broader implementation.

Response: We thank the reviewer for this comment. We have revised the terms in the following:

Results pages 9 and 10, lines 248-265:

“The model predicting depressive symptoms among Lebanese adults retained ten predictors: education, number of chronic illnesses, pain that impact usual activity, household eviction notice, household water insecurity, household food insecurity, debt status, perceived safety inside home, housing tenure status, and number of people to count on in difficult times. This model had an AUC=0.81; 95%CI: 0.78-0.84, C-Slope=1.06; 95% CI: 0.90-1.21, a CITL of -0.002, and E:O of 1.001 (Figure 1 and Supplemental Table 6). Predictors from the model predicting anxiety symptoms among Lebanese adults are presented in Supplemental Table 6. Similarly, the model had an AUC=0.80; 95%CI: 0.77-0.83, C-Slope=1.02; 95%CI: 0.87-1.17, a CITL of 0.004, and E:O of 0.998 (Supplemental Figure 2 and Supplemental Table 6).

The model predicting depressive symptoms among Syrian refugees or migrants retained ten predictors: education, marital status, number of chronic diseases, pain that impact usual activity, household eviction notice, household food insecurity, debt status, perceived safety inside home, number of people to count on in difficult times, and legal residency permit possession. This model had an AUC=0.83; 95%CI: 0.80-0.88, C-Slope=1.02; 95%CI: 0.84-1.19, a CITL of -0.073, and E:O of 1.028 (Figure 2 and Supplemental Table 7). Predictors of the model predicting anxiety symptoms among Syrian refugees or migrants are presented in Supplemental Table 7. Similarly, the model had an AUC=0.72; 95%CI: 0.68-0.76, C-Slope=1.01; 95%CI: 0.79-1.23, a CITL of -0.031, and E:O of 1.014 (Supplemental Figure 3 and Supplemental Table 7).”

We have also added the following to the limitations section:

Discussion page 12, lines 328-329:

“External validation among independent Lebanese and Syrian populations is required before wider use.”

Fifth, please comment more directly on generalisability. The study is well justified as a context-specific analysis of low socioeconomic neighbourhoods in Sin-El-Fil, but the abstract and conclusion should reiterate that these models are most applicable to similar urban, crisis-affected, low-resource settings rather than to all Lebanese or Syrian populations in Lebanon. This point is already acknowledged, but it deserves slightly more prominence because readers may otherwise overextend the findings.

Response: We thank the reviewer for this comment. The following was added in the abstract and conclusion sections:

Abstract *Conclusions* section page, lines:

“These models are most applicable in similar urban, crisis-affected, and low-resource settings.”

Discussion *Conclusion* section page 2, lines 56-57:

“These models are most applicable to similar urban, crisis-affected, low-resource settings.”

I also have a few minor suggestions for the presentation. Please ensure complete consistency in terminology for Syrians, for example, refugees or migrants, across the manuscript.

Please check the wording around legal residency documentation, so the operational definition is immediately clear to an international readership.

In addition, some references used in the discussion appear less authoritative than others for a high-impact public health journal, so I encourage you to review the reference list and, where possible, prioritise peer-reviewed or institutional sources.

Response: We thank the reviewer for this comment. The terminology Syrian refugees or migrants was revised to ensure consistency throughout the whole manuscript, Supplementary Appendix and Table/ Figure titles. Example of the changes made throughout the manuscript include:

Abstract page 2, lines 34-36:

"Objective: This study aimed to develop prediction models for symptoms of poor mental health among Lebanese adults and adult Syrian refugees or migrants residing in a suburb of Beirut, Lebanon, separately."

Abstract page 2, lines 46-58:

Results: Of 1,986 participants, 1,322 (66.5%) were Lebanese adults, 664 (33.5%) were Syrian refugees or migrants. Among Lebanese adults and Syrian refugees or migrants, 324 (25.3%) and 289 (43.9%) had depressive symptoms, respectively. Having pain that impacts usual activity, having debt, not feeling safe at home, and having none or one person to count on in difficult times were common predictors of depressive and anxiety symptoms among Lebanese adults and Syrian refugee or migrants. Not having a legal residency permit was also a predictor of depressive symptoms for Syrian refugees or migrants. Prediction models developed for depressive and anxiety symptoms among both nationalities had good performance measures.

Conclusions: The predictors of poor mental health were financial, health, and social indicators for both Lebanese adults and Syrian refugees or migrants during the concurrent crisis in Lebanon. These models are most applicable in similar urban, crisis-affected, and low-resource settings. Findings emphasise the need for social protection and financial support among population with vulnerabilities.

Strengths and Limitations of This Study page 3, lines 65-66:

"• Replicable depressive and anxiety symptoms' prediction models among Lebanese adults and Syrian refugees or migrants residing in Sin-El-Fil were developed."

Methods *Sampling and study population* section pages 5 and 6, lines 137-145:

"The parent study included participants with specific vulnerabilities from high and low SES areas. In high SES areas, all adult Syrian refugees or migrants, all pregnant adult women,

and all older adults were included. In low SES areas, all adult Syrian refugees or migrants and all pregnant adult women were included, while older adults and adults aged 18–60 years were randomly selected using proportionate allocation. The present study includes only adult participants residing in low SES areas who completed the first wave of data collection conducted between June and October 2022 (n=2,045), and the analysis was restricted to Lebanese adults and adult Syrian refugees or migrants (n=1,986); individuals of any other nationality were excluded [19,20] (Supplemental Figure 1)."

Results page 9, lines 237-239:

"A total of 2,045 respondents participated in this study. The study population after restriction to Lebanese and Syrian nationalities included 1,986 participants of which 1,322 (66.5%) were Lebanese adults and 664 (33.5%) were Syrian refugees or migrants (Supplemental Figure 1)."

Discussion page 10, lines 272-276:

"This study explored predictors of depressive and anxiety symptoms among Lebanese adults and Syrian refugees or migrants residing in low SES areas of Sin-El-Fil. Four prediction models were developed, and their discrimination and calibration were assessed. These models identified common and disparate stressors of depressive and anxiety symptoms among Lebanese adults and Syrian refugees or migrants residing in Sin-El-Fil."

Supplemental Appendix *Results* section page 6, lines 128-129:

"This study explored predictors of depressive and anxiety symptoms among Lebanese adults and Syrian refugees or migrants residing in low SES areas of Sin-El-Fil. Four prediction models were developed, and their discrimination and calibration were assessed. These models identified common and disparate stressors of depressive and anxiety symptoms among Lebanese adults and Syrian refugees or migrants residing in Sin-El-Fil."

We have also included a definition of legal residency permit as the following:

Methods *Candidate predictors* section page 7, lines 183-189:

"Fifteen candidate predictors were included for the development of the depressive and anxiety symptoms prediction models among the Syrian subpopulation. These predictors were: sex, age, education, marital status, number of chronic illnesses, pain that impacts usual activity, household water insecurity, household food insecurity, employment status, debt status, perceived safety inside home, household eviction notice, number of people to count on in difficult times, receipt of cash assistance, and legal residency permit possession characterised by having a government-issued document allowing Syrian refugees or migrants to legally reside in Lebanon."

Finally, due to limited literature on this topic, especially in the Lebanese context, evidence from high-impact journals was not always available. We also included two relevant references to further support our discussion:

- Smith-Clapham AM, Childs JE, Cooley-Strickland M, et al. Implications of the COVID-19 pandemic on interpersonal violence within marginalized communities: Toward a new prevention paradigm. Am J Public Health. 2023;113(S2):S149-S56.

-Devries KM, Mak JY, Bacchus LJ, et al. Intimate partner violence and incident depressive symptoms and suicide attempts: a systematic review of longitudinal studies. PLoS Med. 2013;10(5):e1001439.

VERSION 3 - REVIEW

Reviewer 2

Name Fornaro, Michele

Affiliation Section of Psychiatry, University School of Medicine Federico II, Naples, Italy

Date 31-Mar-2026

COI

Thank you for your revision.
